# SPARC Drives Podocyte Mitochondrial Damage and Ferroptosis in Diabetic Kidney Disease Following Klotho Deficiency

**DOI:** 10.1002/advs.77202

**Published:** 2026-08-29

**Authors:** Qing Yang, Huimin Jiang, Chenyang Xiong, Xindi Zhou, Qinqin Tan, Han Xiao, Lan Qiu, Chun Gan, Yaxi Chen, Haiping Yang, Wei Jiang, Qiu Li

**Affiliations:** ^1^ National Clinical Research Center for Children and Adolescents’ Health and Diseases Ministry of Education Key Laboratory of Child Development and Disorders Chongqing Key Laboratory of Pediatric Metabolism and Inflammatory Diseases Children's Hospital of Chongqing Medical University Chongqing People's Republic of China; ^2^ Centre For Lipid Research & Chongqing Key Laboratory of Metabolism on Lipid and Glucose the Second Affiliated Hospital of Chongqing Medical University Chongqing People's Republic of China

**Keywords:** CUX1, DKD, Klotho, Podocyte Ferroptosis, SPARC

## Abstract

Diabetic kidney disease (DKD) is a leading cause of end‐stage renal disease, with podocyte injury driving disease progression. Mitochondrial dysfunction and ferroptosis contribute to podocyte loss, yet the underlying molecular mechanisms remain incompletely understood. The anti‐aging protein Klotho protects podocytes, but how Klotho deficiency links to mitochondrial dysfunction and ferroptosis is unclear. Here, we identify SPARC, a matricellular protein upregulated in podocytes, as a key mediator linking Klotho deficiency to mitochondrial injury and ferroptosis. In human DKD biopsies and genetically modified mice, including Klotho‐deficient, Klotho‐overexpressing, and SPARC knockout mice, reduced Klotho expression correlated with ferroptosis markers (GPX4, SLC7A11, P53, 4‐HNE, and ACSL4) and mitochondrial abnormalities. Mechanistically, Klotho deficiency activated PKCα, leading to ubiquitination‐mediated degradation of the transcription factor CUX1. Loss of CUX1 derepressed SPARC, which activated TGFβ‐RII/Smad signaling, leading to mitochondrial dysfunction and ferroptosis. In vitro, SPARC knockdown or CUX1 overexpression preserved mitochondrial structure and ferroptosis defenses under high glucose, whereas SPARC overexpression alone induced ferroptosis even in normoglycemic conditions. These findings establish a Klotho/PKCα/CUX1/SPARC/TGFβ‐RII axis as a critical regulator of podocyte mitochondrial dysfunction and ferroptosis. Beyond DKD, SPARC knockout also protected podocytes in ischemia‐reperfusion injury and unilateral ureteral obstruction models, suggesting that SPARC may have broader implications in kidney injury and the progression toward chronic kidney disease.

## Graphical abstract

Klotho deficiency promotes podocyte mitochondrial dysfunction and ferroptosis through activation of the PKCα/CUX1/SPARC/TGFβ‐RII axis. SPARC emerges as a key mediator linking Klotho loss to podocyte injury in DKD and other kidney injury models, suggesting broader implications for CKD progression.

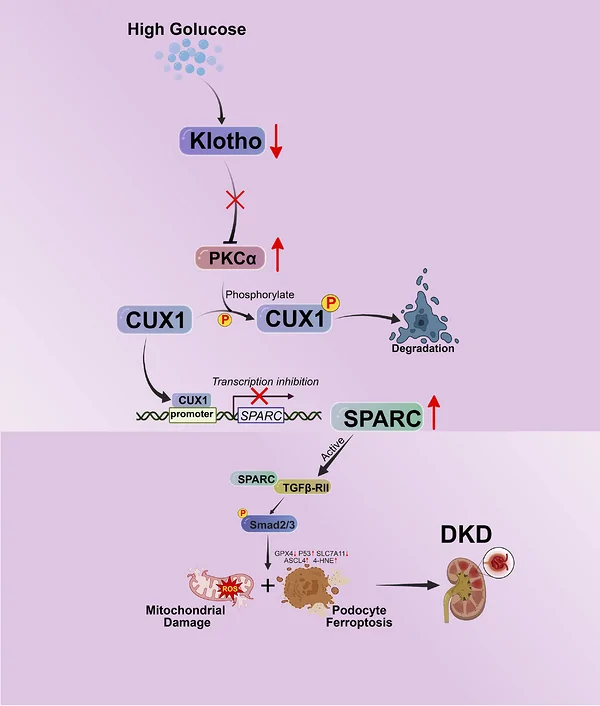

## 1 Introduction

Diabetic kidney disease (DKD) is characterized by a gradual decline in estimated glomerular filtration rate accompanied by progressive proteinuria, making it the leading cause of chronic kidney disease (CKD) and end‐stage renal disease (ESRD), with a significant impact on long‐term mortality in individuals with diabetes mellitus and CKD [1, 2]. Current therapies, including renin‐angiotensin system blockers, sodium‐glucose cotransporter 2 inhibitors, glucagon‐like peptide 1 receptor agonists, and nonsteroidal mineralocorticoid receptor antagonists, can slow disease progression but do not prevent its advancement [3, 4, 5, 6, 7, 8]. Podocytes, highly specialized and terminally differentiated cells, are essential for maintaining the integrity and selectivity of the glomerular filtration barrier, and their unique architecture renders them particularly susceptible to metabolic stress, oxidative injury, and structural damage [2, 9]. In DKD, podocyte injury is the earliest and most critical glomerular event, driving proteinuria and influencing both the rate and severity of disease progression [9, 10]. Thus, elucidating and targeting the mechanisms underlying podocyte injury is pivotal for developing more effective therapies in DKD.

Mitochondria are the primary energy‐generating organelles in podocytes, and their dysfunction is a key driver of podocyte injury in DKD [11]. High glucose toxicity, through multiple aberrant metabolic pathways, induces excessive reactive oxygen species (ROS) production, disrupts mitochondrial dynamics, impairs mitophagy, and reduces mitochondrial biogenesis, ultimately leading to energy depletion, oxidative stress, and cell death [12, 13]. Ferroptosis, an iron‐dependent form of regulated cell death, is characterized by iron overload, lipid peroxidation, and ROS accumulation, with distinct morphological and biochemical features compared to apoptosis or necrosis [14, 15]. In podocytes, ferroptosis is driven by excessive lipid ROS and represents a central determinant of their vulnerability [16]. Recent single‐nucleus RNA sequencing studies have identified podocytes as the primary cellular targets of ferroptosis in DKD, suggesting that elucidating its regulatory network may provide new opportunities for precision therapy. Increasing evidence points to a complex interplay between mitochondrial dysfunction and ferroptosis, with these processes converging to amplify kidney injury [17, 18, 19]. Nevertheless, the precise molecular mechanisms of podocyte injury mediated by a combination of mitochondrial dysfunction and ferroptosis in DKD remain incompletely defined.

α‐Klotho (also referred to as Klotho) is an anti‐aging protein with pleiotropic renoprotective functions, predominantly expressed in the kidney, parathyroid gland, brain, and adipose tissue [20]. Klotho also regulates energy metabolism by modulating the endocrine activity of fibroblast growth factors, including FGF‐23 [21, 22]. Our previous work demonstrated that Klotho could attenuate oxidized low‐density lipoprotein (ox‐LDL)‐induced podocyte injury in DKD, yet evidence indicates that even optimal glycemic and lipid control often fails to prevent progression to ESRD [23, 24], highlighting the complexity and multifactorial nature of podocyte injury in DKD. Given Klotho's broad cytoprotective effects and the unresolved mechanisms of its action, we hypothesized that Klotho may modulate podocyte ferroptosis, a possibility that had not been fully explored before.

The secreted protein acidic and rich in cysteine (SPARC) is a multifunctional, calcium‐binding matricellular glycoprotein that regulates cell adhesion, migration, tissue repair, and remodeling [25, 26, 27]. Beyond these classical roles, SPARC has recently been implicated in the regulation of ferroptosis, particularly in human hepatocellular carcinoma [28]. In this study, we identify SPARC as a crucial mediator linking Klotho deficiency to podocyte injury in DKD. We demonstrate that loss of Klotho triggers a cascade that disrupts mitochondrial function and sensitizes podocytes to ferroptosis. Mechanistically, Klotho deficiency activates the SPARC/TGFβ‐RII/Smad signaling pathway, thereby promoting mitochondrial dysfunction and ferroptosis. Further investigation revealed that the transcription factor CUX1 represses SPARC expression, whereas Klotho deficiency promotes protein kinase Cα (PKCα)‐dependent CUX1 degradation, relieving SPARC suppression. Consequently, elevated SPARC amplifies mitochondrial dysfunction and ferroptosis, accelerating podocyte injury. Furthermore, genetic deletion of SPARC protected against podocyte injury in ischemia‐reperfusion injury (IRI) and unilateral ureteral obstruction (UUO) models, suggesting a broader role for SPARC in kidney injury and progression toward CKD. Collectively, our findings reveal that Klotho downregulation activates a PKCα/CUX1/SPARC regulatory network that links mitochondrial dysfunction and ferroptosis in podocytes, identifying SPARC as a potential therapeutic target for preserving podocyte integrity and slowing DKD progression.

## 2 Results

### 2.1 Klotho Deficiency Exacerbates Podocyte Ferroptosis in DKD

**FIGURE 1:**
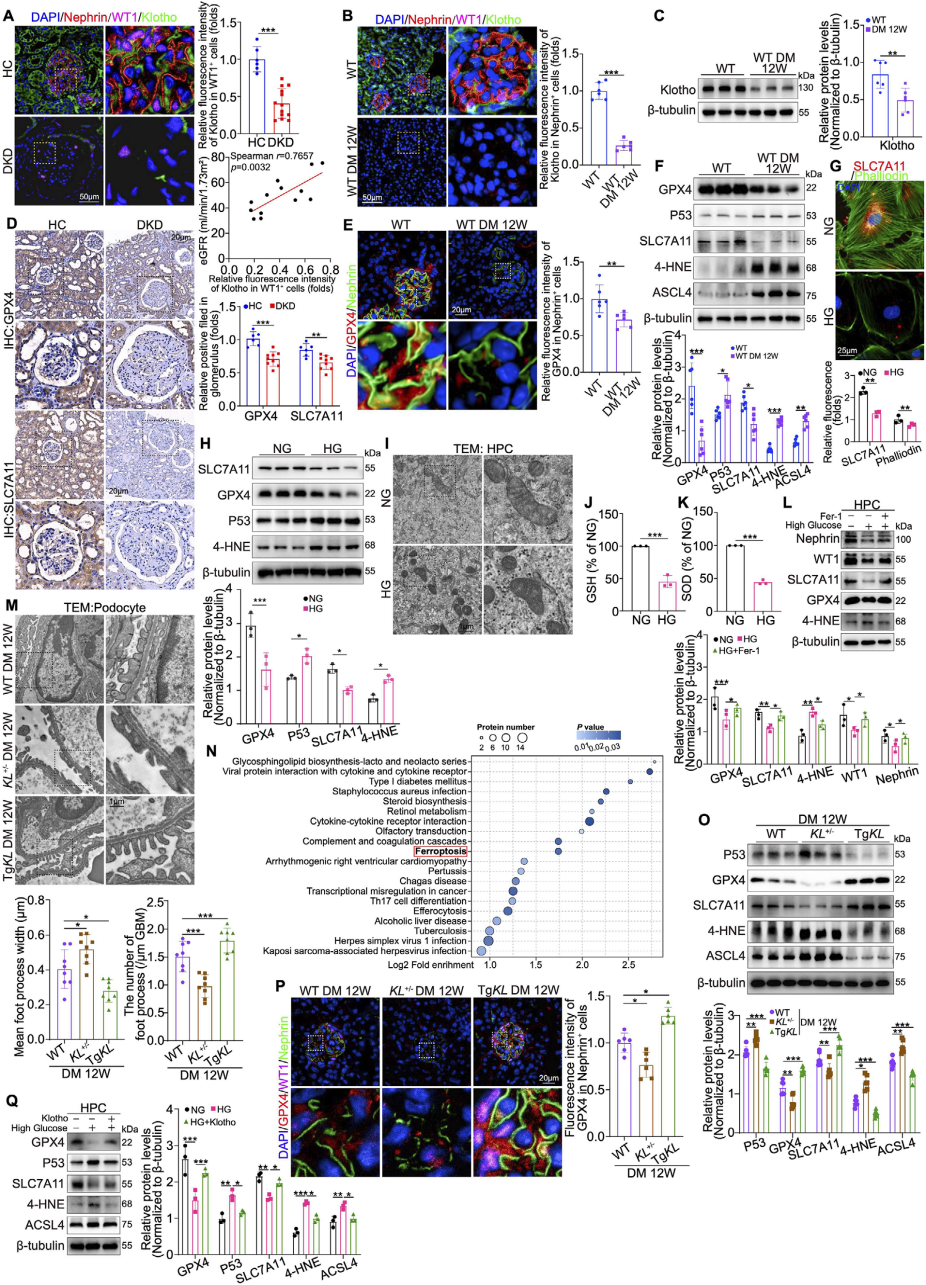
Klotho deficiency exacerbates podocyte ferroptosis in DKD. (A) IF staining for Klotho, Nephrin, and WT1 and quantitative analysis of Klotho expression in podocytes of renal biopsies from healthy controls (n = 6) and DKD patients (*n* = 13). Scale bar, 50 µm. Correlation analysis between the expression of Klotho in podocytes and eGFR (*n* = 13). (B) Representative IF images of Nephrin, WT1, and Klotho and quantitative analysis of Klotho expression in podocytes from control and diabetic mice (*n* = 6). Scale bar, 50 µm. (C) Western blot and quantification of renal Klotho in diabetic mice (*n* = 6). (D) Immunohistochemical staining and quantitative analysis for GPX4 and SLC7A11 in glomeruli of healthy controls (*n* = 6) and DKD patients (*n* = 9). Scale bar, 20 µm. (E) IF for GPX4 and Nephrin and quantitative analysis of GPX4 expression in podocytes of diabetic mice (*n* = 6). Scale bar, 20 µm. (F) Western blot and quantification of GPX4, P53, 4‐HNE, ACSL4, and SLC7A11 in isolated glomeruli from control and diabetic mice (*n* = 6). (G) IF staining and quantitative analysis of SLC7A11 and F‐actin (Phalloidin) in podocytes under high glucose (HG) (*n* = 3). Scale bar, 25 µm. (H) Western blot and quantification of GPX4, P53, 4‐HNE, and SLC7A11 in podocytes exposed to normal glucose and HG (*n* = 3). (I–K) Biochemical assays showing mitochondrial abnormalities by TEM (I, Scale bar, 1 µm), reduced GSH (J), and SOD activity (K) in podocytes under HG (*n* = 3). (L) Quantification and representative western blot of GPX4, Nephrin, WT1, 4‐HNE, and SLC7A11 in podocytes following exposure to HG combined with Ferrostatin‐1 (*n* = 3). (M) Transmission electron micrographs of podocyte ultrastructure and quantification of foot process width and number in WT, *KL* ^+/−^, and Tg*KL* diabetic mice (*n* = 8). Scale bar, 1 µm. (N) Top 20 KEGG pathways enriched in podocytes treated with HG ± recombinant Klotho (400 µM); dot color indicates‐log_10_(P), size reflects gene count. (O) Glomerular Western blot and quantification for GPX4, P53, 4‐HNE, ACSL4, and SLC7A11 in WT, *KL* ^+/−^, and Tg*KL* diabetic mice (*n* = 6). (P) Co‐IF and quantification for GPX4 in podocytes from WT, *KL* ^+/−^, and Tg*KL* diabetic mice (*n* = 6). Scale bar, 20 µm. (Q) Western blot and quantification of GPX4, P53, 4‐HNE, ACSL4, and SLC7A11 in podocytes treated with HG ± recombinant Klotho. Data are mean ± SD; ^*^ *P* < 0.05, ^**^ *P* < 0.01, ^***^ *P* < 0.001, One‐way ANOVA with Dunnett's test or Tukey's test (L, M, O–Q), Pearson's correlation test (A), unpaired Student's *t*‐test (A–H, J and K). IF, Immunofluorescence; TEM, Transmission electron microscopy; HG, high glucose; WT, Wild‐Type.

Building on our prior evidence of Klotho's protective role in DKD [23, 29], we first assessed its expression in human renal tissue. Immunofluorescence (IF) staining of glomeruli from DKD patient biopsies revealed markedly reduced Klotho levels compared to healthy controls (HC), which showed a positive correlation with estimated glomerular filtration rate (eGFR) (Figure 1A). To model DKD in vivo, we induced diabetes in mice using streptozotocin combined with a high‐fat diet (STZ/HFD). By 12 weeks post‐induction, diabetic mice exhibited fasting blood glucose levels exceeding 16.7 mM, renal dysfunction with elevated Blood urea nitrogen (BUN), serum creatinine, and urinary albumin‐to‐creatinine ratio (UACR) levels, and classic DKD histopathology, including mesangial expansion and glomerular glycogen accumulation, as confirmed by hematoxylin–eosin (HE) and Periodic Acid–Schiff (PAS) staining (Figure S1A,B). Transmission electron microscopy (TEM) further demonstrated ultrastructural glomerular injury, characterized by podocyte foot process effacement and thickening of the glomerular basement membrane (Figure S1C). At this timepoint, both immunohistochemistry (IHC) and IF staining showed significantly diminished glomerular Klotho expression in WT DM 12 W mice relative to WT mice, a finding corroborated by Western blot analysis (Figure 1B,C and Figure S1D).

To assess ferroptosis in podocytes under DKD conditions, we examined the expression of the core ferroptosis regulators GPX4 and SLC7A11 in human renal biopsies. Although ferroptosis signals were strongest in tubules, glomeruli of DKD patients also exhibited reduced GPX4 and SLC7A11 (Figure 1D). In diabetic mice at 12 weeks, podocyte‐specific IF staining confirmed decreased GPX4 and increased 4‐HNE in glomeruli (Figure 1E and Figure S1E). Western blot analysis of glomerular lysates further revealed downregulation of both GPX4 and SLC7A11, alongside upregulation of P53, a transcriptional repressor of SLC7A11, as well as increased 4‐HNE and ACSL4 (Figure 1F). To validate these observations in a controlled setting, we exposed cultured podocytes to high glucose (HG). Consistent results were observed in vitro, where cultured podocytes exposed to HG exhibited reduced SLC7A11 expression by IF, as well as suppressed GPX4 and SLC7A11 and increased P53 and 4‐HNE at the protein level by Western blot (Figure 1G,H). TEM revealed mitochondrial shrinkage, a hallmark of ferroptosis, in HG‐treated podocytes (Figure 1I). These cells also displayed significant depletion of glutathione (GSH) and reduced superoxide dismutase (SOD) activity (Figure 1J,K). Treatment with the ferroptosis inhibitor Ferrostatin‐1 (Fer‐1) reversed HG‐induced changes, restoring GPX4, SLC7A11, Nephrin, and WT1 expression while reducing 4‐HNE and Fe^2+^ accumulation (Figure 1L and Figure S1F).

To directly interrogate Klotho's role, Klotho‐deficient (*KL* ^+/−^) and Klotho‐overexpressing (Tg*KL*) mice were subjected to the diabetic model (Figure S1G). At 12 weeks, *KL* ^+/−^ mice exhibited more severe renal dysfunction and glomerular pathology, including pronounced mesangial proliferation and glycogen deposition, whereas Klotho overexpression mitigated these structural abnormalities (Figure S1H,I). TEM confirmed that Klotho deficiency worsened podocyte foot process effacement and basement membrane thickening, while Klotho overexpression preserved podocyte architecture (Figure 1M). Tandem mass spectrometry of podocytes exposed to HG identified ferroptosis as a major pathway modulated by Klotho (Figure 1N). Western blot analysis of glomerular tissues showed that Klotho suppressed P53, ACSL4, and 4‐HNE expression while enhancing SLC7A11 and GPX4 levels (Figure 1O). Podocyte‐specific IF confirmed restoration of GPX4 and reduction of 4‐HNE in vivo (Figure 1P and Figure S1J). In vitro, Klotho overexpression similarly inhibited P53, ACSL4, and 4‐HNE while upregulating SLC7A11 and GPX4 (Figure 1Q and Figure S1K). Functionally, Klotho increased GSH, SOD, and ATP levels, ameliorated mitochondrial shrinkage, reduced Fe^2+^ accumulation, and suppressed lipid peroxidation, as evidenced by decreased C11‐BODIPY oxidation under high‐glucose conditions (Figure S1L–N). Taken together, these findings demonstrate that Klotho deficiency aggravates podocyte ferroptosis in DKD, whereas Klotho supplementation effectively alleviates it.

### 2.2 Klotho Attenuates Mitochondrial Dysfunction

**FIGURE 2:**
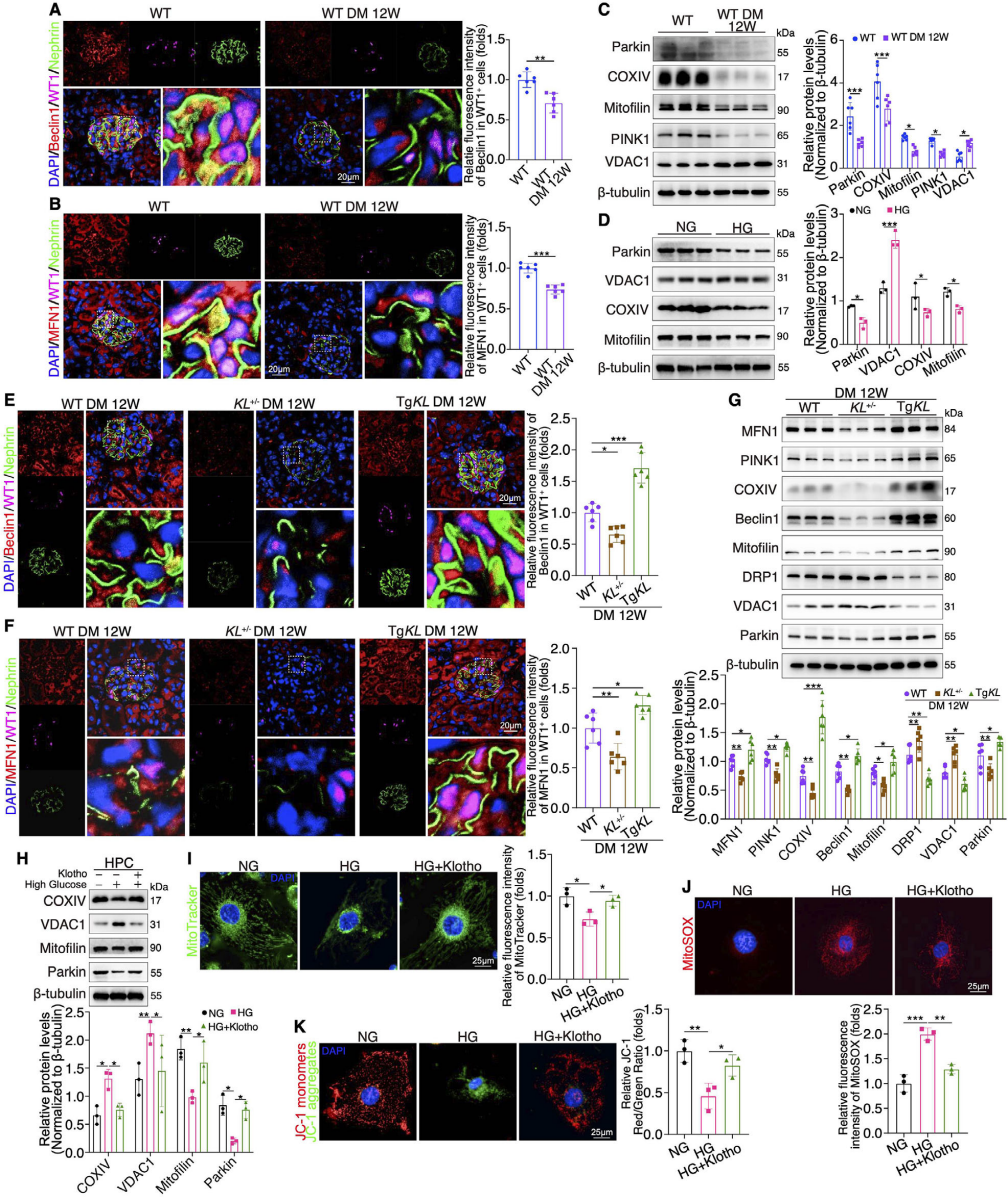
Klotho attenuates mitochondrial dysfunction. (A, B) IF staining for Beclin1, Nephrin, WT1, and MFN1 and quantitative analysis of Beclin1, MFN1 in podocytes of control and diabetic mice (*n* = 6). Scale bar, 20 µm. (C) Western blot and quantitative analysis of mitochondrial proteins (Parkin, PINK1, COXIV, Mitofilin, VDAC1) in glomerular lysates from control and diabetic mice (*n* = 6). (D) Western blot and quantitative analysis showing the effect of HG on Parkin, VDAC1, COXIV, and Mitofilin in cultured podocytes (*n* = 3). (E, F) Glomerular co‐IF staining for Beclin1, MFN1, Nephrin, and WT1 and quantitative analysis of Beclin1, MFN1 in podocytes of WT, *KL* ^+/−^, and Tg*KL* diabetic mice at 12 weeks post‐induction (*n* = 6). Scale bar, 20 µm. (G) Western blot and quantification of mitochondrial dynamics and mitophagy markers (MFN1, DRP1, PINK1, Parkin, Beclin1, COXIV, VDAC1, Mitofilin) in glomerular tissue from WT, *KL* ^+/−^, and Tg*KL* diabetic mice (*n* = 6). (H) Protein levels of COXIV, VDAC1, Mitofilin, and Parkin in podocytes treated with HG ± recombinant Klotho (*n* = 3). (I–K) Representative images and quantitative analysis of mitochondrial mass (MitoTracker, I), mitochondrial superoxide production (MitoSOX, J), and membrane potential (JC‐1, K) in podocytes under indicated treatments (*n* = 3). Scale bar, 25 µm. Data are mean ± SD; ^*^ *p* < 0.05, ^**^ *p* < 0.01, ^***^ *p* < 0.001, One‐way ANOVA with Dunnett's test or Tukey's test (E–K), unpaired Student's *t*‐test (A–D). IF, Immunofluorescence; HG, high glucose; WT, Wild‐Type.

Given the well‐established link between ferroptosis and mitochondrial dysfunction in oxidative stress‐related injury, we next evaluated mitochondrial integrity. Using IF staining, we assessed the expression of two mitochondrial quality control proteins, Beclin1 and MFN1, and found both to be significantly downregulated in podocytes of diabetic mice (Figure 2A,B). These observations were further confirmed by Western blot analysis, which showed reduced levels of key mitochondrial proteins, including Parkin, COXIV, Mitofilin and PINK1, along with increased expression of VDAC1 (Figure 2C). In vitro, HG stimulation of cultured podocytes recapitulated these changes, with decreased expression of Parkin, COXIV and Mitofilin, alongside elevated VDAC1 levels (Figure 2D).

We next examined the impact of Klotho on mitochondrial function. IF staining analysis of podocytes from WT, *KL* ^+/−^ and Tg*KL* mice at week 12 post‐diabetes induction revealed that Klotho deficiency further suppressed Beclin1 and MFN1 expression, whereas Klotho overexpression significantly alleviated this suppression (Figure 2E,F). Western blot analysis of glomerular tissues confirmed that Klotho enhanced the expression of mitochondrial‐associated proteins, including MFN1, PINK1, COXIV, Beclin1, Mitofilin and Parkin, while suppressing DRP1 and VDAC1 (Figure 2G). Similarly, in HG‐treated podocytes in vitro, Klotho overexpression increased the levels of COXIV, Mitofilin and Parkin and reduced VDAC1 expression (Figure 2H). Furthermore, Klotho restored mitochondrial morphology and attenuated fragmentation in podocytes exposed to HG, as demonstrated by MitoTracker staining. It also significantly suppressed mitochondrial ROS production, as shown by reduced MitoSOX fluorescence, and preserved mitochondrial membrane potential, as evidenced by JC‐1 staining (Figure 2I–K). These findings indicate that Klotho maintains both structural and functional mitochondrial integrity in podocytes and mitigates ferroptosis in DKD.

### 2.3 Klotho Alleviates Mitochondrial Dysfunction and Ferroptosis by Inhibiting SPARC

**FIGURE 3:**
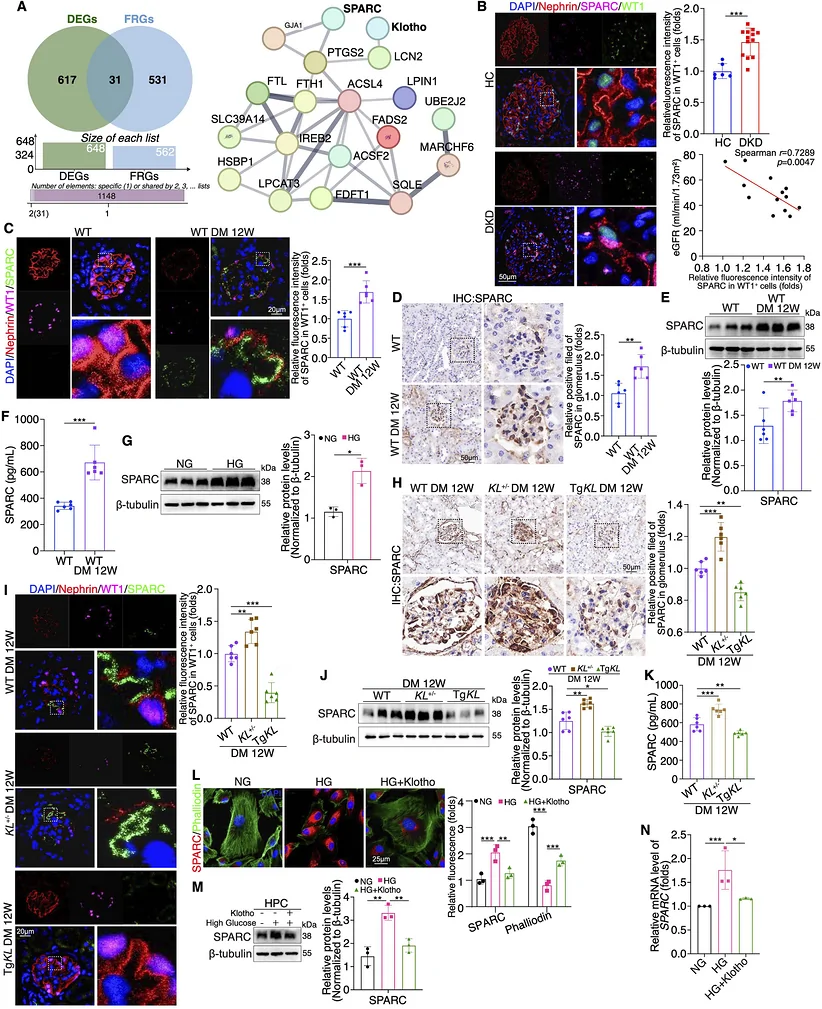
Klotho regulates SPARC expression in podocytes. (A) Venn diagram showing the overlap between differentially expressed genes identified by tandem mass spectrometry and ferroptosis‐related genes, followed by STRING‐based protein–protein interaction network analysis. (B, C) Glomerular co‐IF staining for SPARC, Nephrin, and WT1 and quantitative analysis of SPARC in podocytes in renal biopsy specimens from DKD patients (B, *n* = 13) and in glomeruli of diabetic mice (C, *n* = 6). Correlation analysis between the expression of SPARC in podocytes and eGFR (*n* = 13). Scale bar, 50 µm (B), 20 µm (C). (D, E) Glomerular SPARC protein levels assessed by IHC staining (D) and Western blot (E) across mouse groups. (*n* = 6). Scale bar, 50 µm. (F) Serum SPARC concentrations in control versus diabetic mice, measured by ELISA (*n* = 6). (G) Western blot and quantification of SPARC in podocytes exposed to HG (*n* = 3). (H, I) Representative images and quantitative analysis of glomerular SPARC protein levels assessed by IHC (H) and IF (I) staining in WT, *KL* ^+/−^, and Tg*KL* diabetic mice (*n* = 6). Scale bar, 50 µm (H), 20 µm (I). (J) Glomerular SPARC protein levels in the different diabetic mouse groups by Western blot (*n* = 6). (K) Serum SPARC levels in WT, *KL* ^+/−^, and Tg*KL* diabetic mice, measured by ELISA (*n* = 6). (L, M) Co‐IF staining (L) and Western blot (M) of SPARC in podocytes treated with HG ± Klotho (*n* = 3). Scale bar, 25 µm. (N) qPCR analysis of *SPARC* mRNA levels in podocytes under the indicated treatments (*n* = 3). Data are mean ± SD; ^*^ *p* < 0.05, ^**^ *p* < 0.01, ^***^ *p* < 0.001, One‐way ANOVA with Dunnett's test or Tukey's test (H–N), Pearson's correlation test (B), unpaired Student's *t*‐test (B–G). IF, Immunofluorescence; ELISA, Enzyme‐Linked Immunosorbent Assay; IHC, Immunohistochemistry; HG, high glucose; WT, Wild‐Type.

To explore downstream mediators, we integrated tandem mass spectrometry with ferroptosis‐related genes from GeneCards and literature [30], identifying SPARC as a candidate effector in podocytes (Figure 3A). IF staining of kidney biopsies revealed significantly elevated SPARC specifically in podocytes of patients with DKD, which was negatively associated with eGFR (Figure 3B). This finding was recapitulated in diabetic mice at 12 weeks, as confirmed by IF, IHC, and Western blot analysis (Figure 3C–E). ELISA also detected markedly elevated circulating SPARC in peripheral blood (Figure 3F). HG treatment of cultured podocytes similarly increased SPARC expression (Figure 3G). Analysis of WT, *KL* ^+/−^, and Tg*KL* mice at week 12 post‐diabetes induction revealed an inverse correlation between Klotho and SPARC expression at tissue and systemic levels, confirmed by IHC, IF, Western blot, and plasma measurement (Figure 3H–K). Critically, recombinant Klotho suppressed HG‐induced SPARC upregulation at the transcriptional level (Figure 3L–N).

**FIGURE 4:**
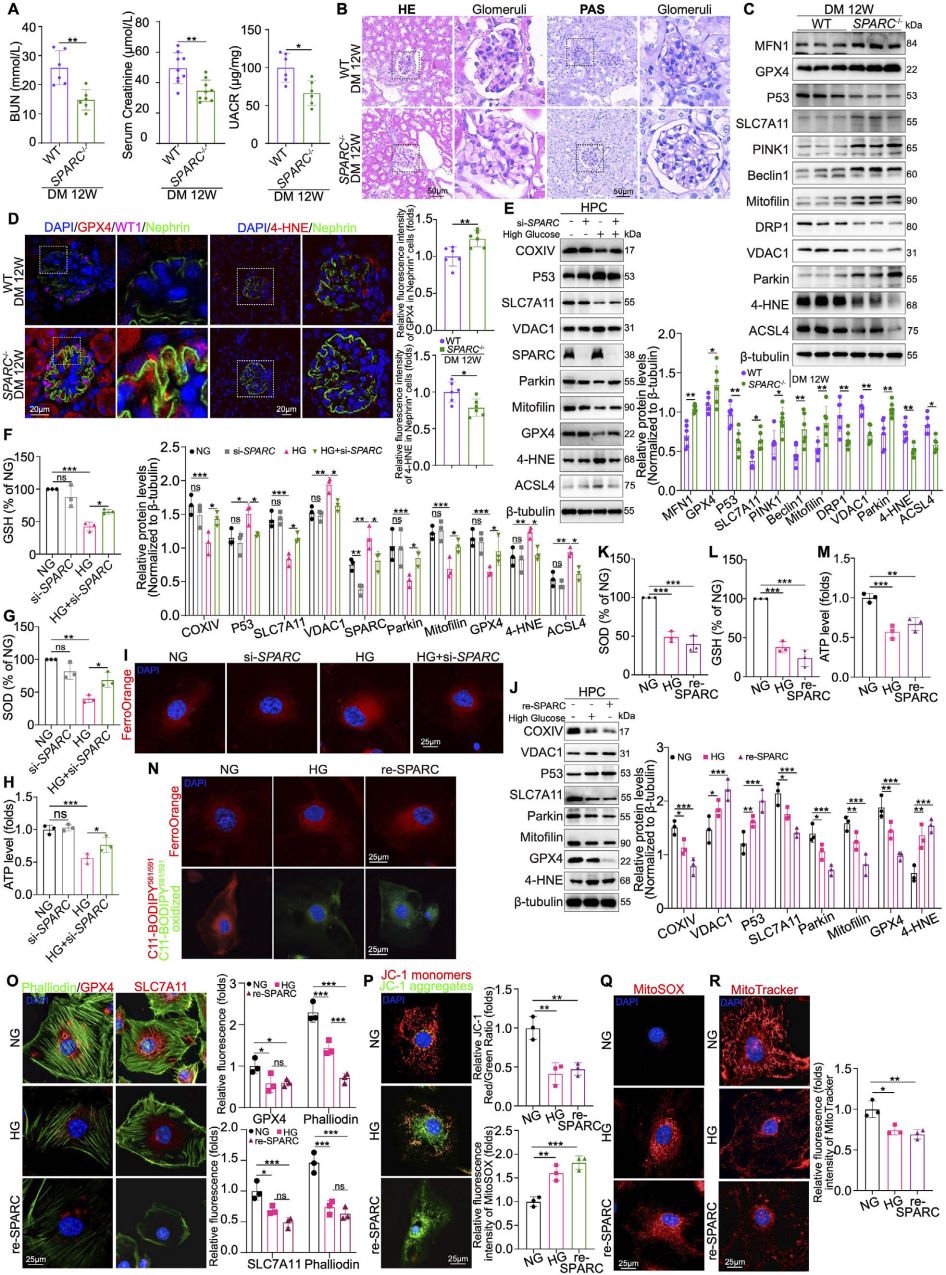
Klotho alleviates mitochondrial damage‐mediated ferroptosis by inhibiting SPARC in DKD. (A) Blood urea nitrogen (BUN), serum creatinine, and urinary albumin‐to‐creatinine ratio (UACR) were measured in WT and *Sparc* ^−/−^ diabetic mice (*n* = 6‐9). (B) Representative HE and PAS staining of kidney sections from WT and *SPARC* ^−/−^ diabetic mice (*n* = 6). Scale bar, 50 µm. (C) Western blot and quantification of MFN1, GPX4, P53, SLC7A11, PINK1, Beclin1, Mitofilin, DRP1, VDAC1, 4‐HNE, ACSL4, and Parkin in glomerular tissue from WT and *SPARC* ^−/−^ diabetic mice (*n* = 6). (D) IF staining of GPX4, 4‐HNE, Nephrin, and WT1 and quantitative analysis of GPX4 and 4‐HNE in podocytes in indicated mouse groups (*n* = 6). Scale bar, 20 µm. (E) Western blot and quantitative analysis of mitochondrial and ferroptosis‐related proteins (COXIV, VDAC1, Mitofilin, Parkin, 4‐HNE, P53, GPX4, SLC7A11, SPARC, ACSL4) in podocytes subjected to various treatments (*n* = 3). (F–H) Suppression of SPARC under HG conditions significantly increased GSH levels (F), SOD activity (G), and ATP levels (H) (*n* = 3). (I) FerroOrange fluorescence intensity reflected the relative content of ferrous ions in each group (*n* = 3). Scale bar, 25 µm. (J) Western blot shows the expression of COXIV, Parkin, VDAC1, Mitofilin, GPX4, SLC7A11, 4‐HNE, and P53, along with quantitative analysis, in podocytes treated with HG or recombinant SPARC protein (*n* = 3). (K–M) SPARC significantly decreased SOD activity (K), GSH levels (L), and ATP levels (M) in experimental groups (*n* = 3). (N–R) IF detection of ferrous ion, C11‐BODIPY^581/591^, ferroptosis regulators (GPX4, SLC7A11) and mitochondrial markers (MitoTracker, JC‐1, MitoSOX) in podocytes under indicated treatments (*n* = 3). Scale bar, 25 µm. Data are presented as mean ± SD; ns, not significant; ^*^ *p* < 0.05, ^**^ *p* < 0.01, ^***^ *p* < 0.001, One‐way ANOVA with Dunnett's test or Tukey's test (E–H, J–M, O–R), unpaired Student's *t*‐test (A, C, D). IF, Immunofluorescence; HG, high glucose; WT, Wild‐Type.

To determine the functional role of SPARC in podocyte ferroptosis, we generated *SPARC* knockout (*SPARC* ^−/−^) mice and subjected them to the STZ/HFD model. SPARC deletion improved renal function, reducing BUN, serum creatinine, and UACR levels, and attenuated glomerular mesangial proliferation and glycogen deposition (Figure 4A,B). Western blot of isolated glomeruli revealed that SPARC silencing restored expression of mitochondrial and antioxidant proteins, including MFN1, GPX4, SLC7A11, PINK1, Beclin1, Mitofilin, and Parkin, while suppressing P53, DRP1, VDAC1, 4‐HNE, and ACSL4 (Figure 4C). IF further confirmed enhanced GPX4 expression and reduced 4‐HNE in podocytes (Figure 4D). In vitro, SPARC knockdown in HG‐treated podocytes similarly restored COXIV, SLC7A11, Parkin, Mitofilin and GPX4, while suppressing P53, VDAC1, 4‐HNE and ACSL4 (Figure 4E). Functionally, this was accompanied by increased intracellular GSH, SOD activity and ATP levels, together with reduced Fe^2+^ accumulation (Figure 4F–I and Figure S2A). SPARC interference preserved mitochondrial membrane potential (JC‐1), reduced mitochondrial ROS (MitoSOX), and mitigated mitochondrial fragmentation (MitoTracker). Importantly, it also rescued podocyte cytoskeletal integrity, ferroptosis markers, and lipid peroxidation (Figure S2B). Conversely, SPARC overexpression under normal glucose conditions recapitulated the high‐glucose phenotype, resulting in downregulation of COXIV, Parkin, Mitofilin, SLC7A11 and GPX4, and upregulation of VDAC1, P53 and 4‐HNE. This was accompanied by depletion of GSH, SOD and ATP, increased Fe^2+^ and lipid peroxidation, disruption of cytoskeletal architecture, loss of mitochondrial membrane potential, and elevated mitochondrial ROS with enhanced mitochondrial fragmentation (Figure 4J–R and Figure S2C). Collectively, these results demonstrate that Klotho could alleviate mitochondrial dysfunction and ferroptosis by inhibiting SPARC.

To assess the broader pathophysiological relevance of SPARC, we also examined two nondiabetic kidney injury models, IRI and UUO. By day 14, both models induced renal dysfunction and glomerular abnormalities, including mesangial proliferation and GBM thickening, accompanied by elevated SPARC expression in podocytes as evidenced by IHC and IF staining (Figure S2D–G). These lesions were significantly attenuated in *SPARC* ^−/−^ IRI and UUO mice (Figure S2H–J). Importantly, *SPARC* deletion restored podocyte GPX4 levels, as shown by podocyte‐focused IF staining, and enhanced glomerular expression of Beclin1 and MFN1 (Figure S3A–D). Western blot of isolated glomerulus further confirmed that *SPARC* knockout improved expression of GPX4, Parkin, Mitofilin, and COXIV while reducing 4‐HNE levels (Figure S3E,F). Given the high metabolic demand of proximal tubular epithelial cells (PTECs), we also assessed ferroptosis in this compartment and found that SPARC knockout ameliorated ferroptosis in PTECs as shown by the GPX4 and SLC7A11 IF staining (Figure S3G,H). Together, these findings indicate that SPARC may play a broader role in regulating mitochondrial dysfunction and ferroptosis in renal cells under stress conditions.

### 2.4 Klotho Inhibits SPARC/TGFβ‐RII/Smad‐Driven Mitochondrial Damage and Ferroptosis

**FIGURE 5:**
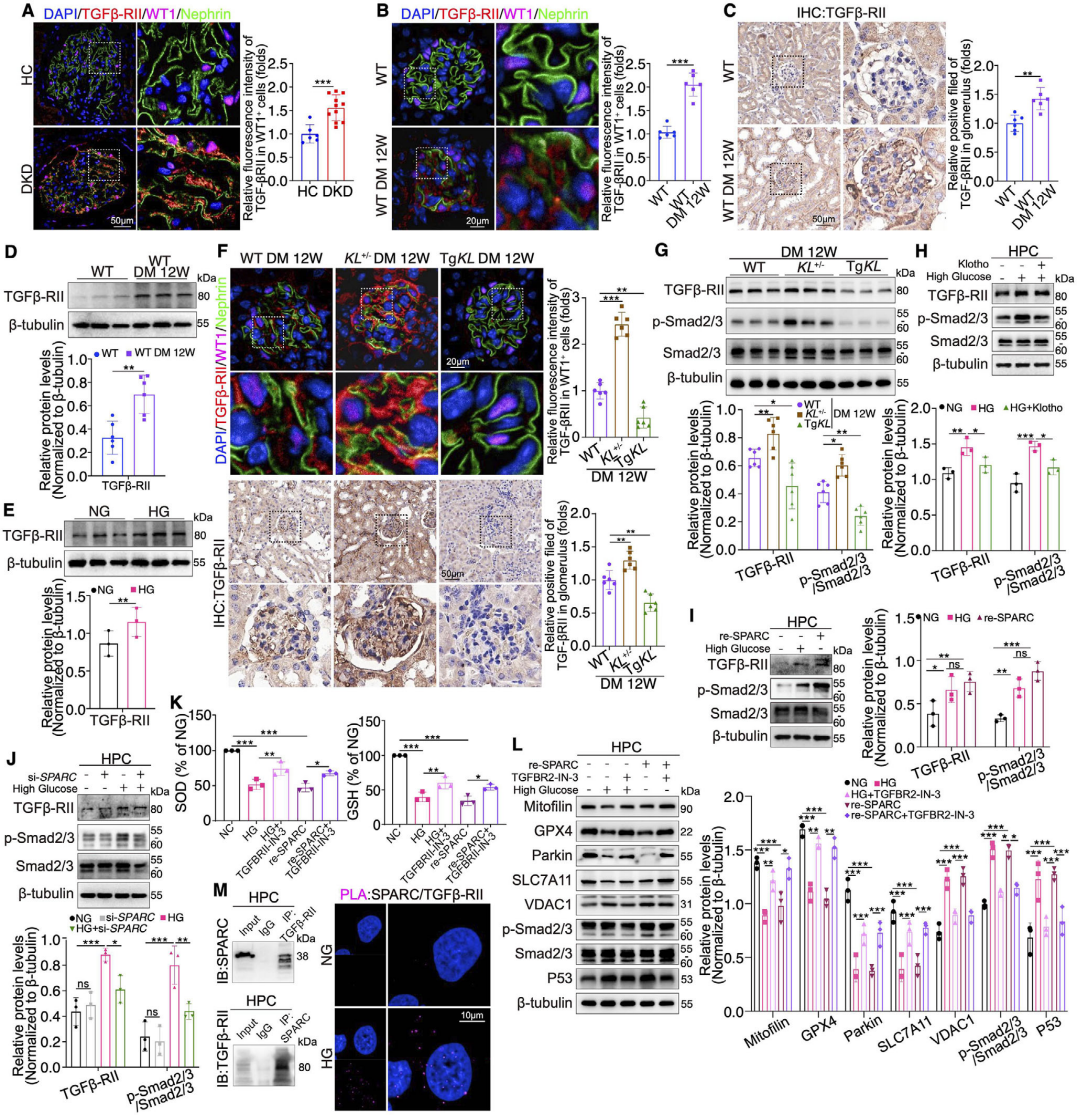
Klotho inhibits SPARC/TGFβ‐RII/Smad‐driven ferroptosis in podocytes. (A, B) Glomerular co‐IF staining for TGFβ‐RII, Nephrin, and WT1 and quantitative analysis of TGFβ‐RII in podocytes in DKD patients (A, *n* = 11) and diabetic mice (B, *n* = 6). Scale bar, 50 µm (A), 20 µm (B). (C, D) Glomerular TGFβ‐RII protein levels assessed by IHC staining (C), and Western blot (D) across experimental mouse groups (*n* = 6). Scale bar, 50 µm (C). (E) TGFβ‐RII expression and quantification in podocytes exposed to HG (*n* = 3). (F) Representative IF and IHC staining combined with IHC staining and quantitative analysis of TGFβ‐RII in WT, *KL* ^+/−^, and Tg*KL* diabetic mice (*n* = 6). Scale bar, 20 or 50 µm. (G) Western blot and quantification of TGFβ‐RII, phosphorylated (p‐) and total Smad2/3 in isolated glomeruli of WT, *KL* ^+/−^, and Tg*KL* diabetic mice (*n* = 6). (H–J) Expression of TGFβ‐RII, p‐Smad2/3, and total Smad2/3 in podocytes under indicated treatments, analyzed by Western blot (*n* = 3). (K) The levels of GSH and the activity of SOD were determined in podocytes among different treatment groups (*n* = 3). (L) The protein levels of p‐Smad2/3, and total Smad2/3, mitochondrial functional markers (Mitofilin, Parkin, VDAC1) and ferroptosis‐related regulators (GPX4, SLC7A11, P53) were determined by Western blot in podocytes of each group (*n* = 3). (M) Co‐immunoprecipitation (Co‐IP) assay and Proximity Ligation Assay confirming the physical interaction between SPARC and TGFβ‐RII in podocytes. Scale bar, 10 µm. Data are presented as mean ± SD; ns, not significant; ^*^ *p* < 0.05, ^**^ *p* < 0.01, ^***^ *p* < 0.001, One‐way ANOVA with Dunnett's test or Tukey's test (F–L), unpaired Student's *t*‐test (A–E). IF, Immunofluorescence; IHC, Immunohistochemistry; HG, high glucose; WT, Wild‐Type.

Although SPARC has been shown to promote podocyte ferroptosis and mitochondrial dysfunction, the precise molecular mechanisms remain incompletely understood. Recent evidence suggests that SPARC may interact with TGFβ receptors to modulate Smad signaling [31, 32]. To explore this in the context of DKD, we performed podocyte‐targeted IF staining on renal biopsies from DKD patients. Compared to HC, TGFβ‐RII was markedly upregulated and prominently localized along podocyte foot process junctions in DKD glomeruli (Figure 5A). A similar pattern was observed in glomeruli of STZ/HFD mice at week 12 post‐induction, further confirmed by IHC and Western blotting of both glomerular lysates and HG‐treated podocytes (Figure 5B–E). To determine whether TGFβ‐RII upregulation in DKD correlates with Klotho loss, we compared glomerular TGFβ‐RII expression across WT, *KL* ^+/−^, and Tg*KL* mice at week 12 post‐diabetes using podocyte‐targeted IF staining. Strikingly, Klotho deficiency further amplified TGFβ‐RII signal intensity, particularly at podocyte foot process junctions, relative to diabetic WT controls (Figure 5F). In contrast, Klotho overexpression markedly suppressed TGFβ‐RII accumulation in the same subcellular compartment (Figure 5F). Additional Western blot analyses of glomerular tissues from these mouse models and HG‐treated podocytes revealed that Klotho expression inversely correlated with activation of the TGFβ‐RII/Smad signaling axis (Figure 5G,H). Specifically, Klotho overexpression attenuated, while Klotho deficiency enhanced, phosphorylation of Smad2/3 downstream of TGFβ‐RII.

To test whether SPARC activates this pathway, we manipulated its expression in podocytes. SPARC overexpression activated TGFβ‐RII/Smad signaling, whereas SPARC knockdown suppressed it (Figure 5I,J). Treatment with a TGFβ‐RII‐specific inhibitor alleviated HG‐ and SPARC‐induced downregulation of Mitofilin, Parkin, GPX4, and SLC7A11, reduced Smad2/3 phosphorylation, P53 and VDAC1, increased SOD activity, and elevated intracellular GSH (Figure 5K,L). Finally, Co‐IP and proximity ligation assay (PLA) in HPC cells confirmed a direct physical interaction between SPARC and TGFβ‐RII (Figure 5M). These data demonstrate that SPARC could bind TGFβ‐RII to activate Smad signaling, thereby driving podocyte mitochondrial damage and ferroptosis.

### 2.5 CUX1 Acts as a Transcriptional Repressor of SPARC

**FIGURE 6:**
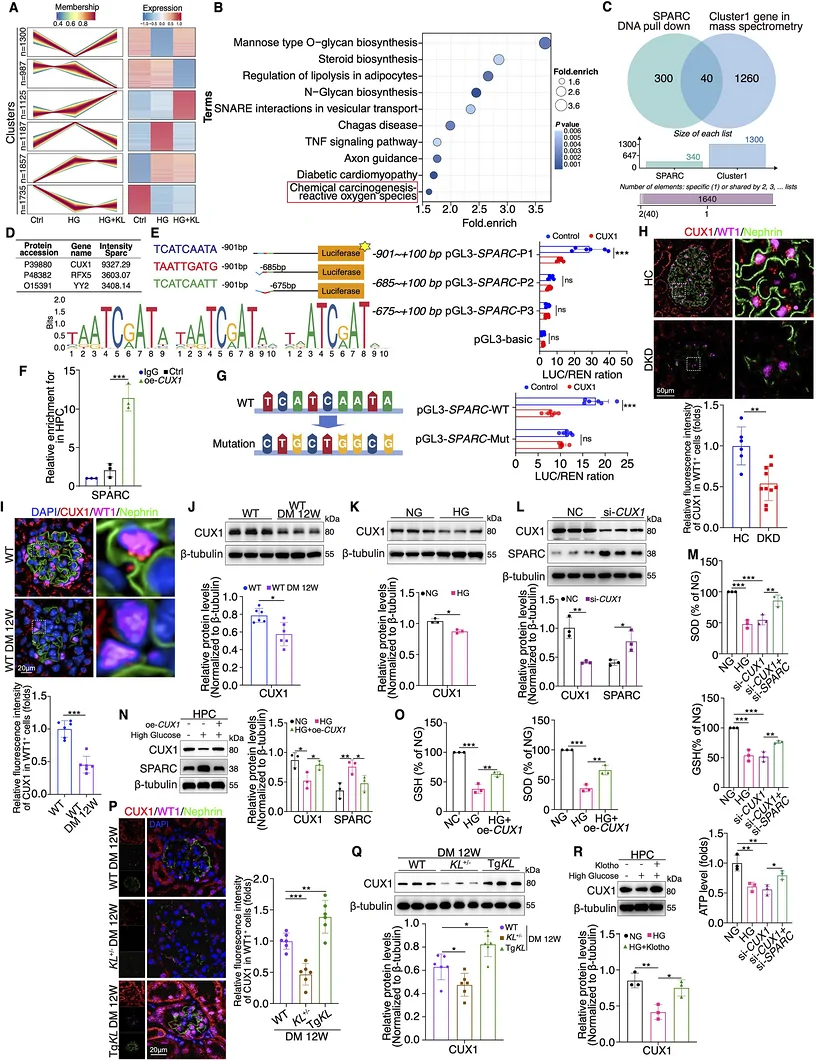
CUX1 acts as a transcriptional repressor of SPARC in podocytes. (A) Heatmap and hierarchical clustering of differentially expressed proteins in podocytes treated with HG ± recombinant Klotho. (B) Top 10 significantly enriched pathways among proteins downregulated by HG; this downregulation was reversed by Klotho pretreatment. (C) Venn diagram showing overlap between SPARC DNA pull‐down‐associated proteins and Cluster 1 differentially expressed proteins identified by mass spectrometry. (D) Top three most abundant proteins in the overlapping set, ranked by spectral intensity. (E, F) Prediction of CUX1 binding sites in the SPARC promoter using JASPAR, validated by dual‐luciferase reporter (*n* = 6) and CUT‐tag analysis (*n* = 3). (G) The inhibitory effect was completely abrogated by mutation of the core binding site, as demonstrated by dual‐luciferase assay (*n* = 6). (H, I) Co‐IF staining for CUX1, Nephrin, and WT1 and quantitative analysis of CUX1 in podocytes in kidney sections from DKD patients (*n* = 10) and indicated mouse groups (*n* = 6). Scale bar, 50 µm (H), 20 µm (I). (J, K) Western blot and quantification analysis of CUX1 protein levels in corresponding mouse (J, *n* = 6) and cellular models (K, *n* = 3). (L) Knockdown of CUX1 increased SPARC expression, confirmed by Western blot (*n* = 3). (M) Combined CUX1 knockdown and SPARC silencing significantly restored SOD activity, GSH levels, and ATP levels in podocytes under HG (*n* = 3). (N) CUX1 overexpression reduced SPARC protein levels in podocytes (*n* = 3). (O) CUX1 overexpression exacerbated the HG‐induced decline in GSH and SOD levels (*n* = 3). (P‐R) IF staining (P, *n* = 6) and Western blot (Q, *n* = 6, R, *n* = 3) analyses reveal a positive correlation between CUX1 and Klotho expression levels. Scale bar, 20 µm (P). Data are presented as mean ± SD; ns, not significant; ^*^ *P* < 0.05, ^**^ *P* < 0.01, ^***^ *P* < 0.001, One‐way ANOVA with Dunnett's test or Tukey's test (F, M‐R), unpaired Student's *t*‐test (E, G‐L). IF, Immunofluorescence; HG, high glucose; WT, Wild‐Type.

SPARC activates TGFβ‐RII/Smad signaling to drive podocyte mitochondrial dysfunction and ferroptosis, positioning it as a key molecular bridge between Klotho deficiency and podocyte injury in DKD. Since Klotho suppresses SPARC primarily at the transcriptional level, we hypothesized that transcriptional regulators mediate this effect. To identify candidate factors, we integrated tandem mass spectrometry with bioinformatic analyses. Heatmap clustering, quantitative protein profiling, and KEGG enrichment revealed that Cluster 1, consisting of proteins downregulated under HG, was enriched for oxidative stress‐related pathways (Figure 6A,B). DNA pull‐down using the SPARC promoter intersected with Cluster 1 identified CUX1, RFX5, and YY2 as top candidates (Figure 6C,D). JASPAR motif analysis revealed three consensus CUX1 binding sites within the SPARC promoter, implicating CUX1 as a direct transcriptional regulator (Figure 6D). Functional validation using dual‐luciferase assays and CUT&Tag‐qPCR confirmed that CUX1 binds a core consensus motif (TCATCAATA) within the −901 to −685 region of the SPARC promoter and represses transcription (Figure 6E,F). Mutation of this motif abolished repression, confirming its essential role (Figure 6G). IF staining of human DKD biopsies and diabetic mice at week 12 revealed reduced CUX1 expression in podocytes, confirmed by IHC and Western blot (Figure 6H–J and Figure S4A). HG treatment in vitro similarly decreased CUX1 (Figure 6K).

CUX1 knockdown elevated SPARC and reproduced HG‐induced mitochondrial and ferroptotic changes, including reduced COXIV, GPX4, Mitofilin and Parkin, increased P53, SPARC, VDAC1, 4‐HNE and ACSL4, decreased GSH and SOD activity, ATP levels, loss of mitochondrial membrane potential (JC‐1), increased mitochondrial ROS (MitoSOX), mitochondrial fragmentation (MitoTracker), elevated intracellular iron (FerroOrange) and lipid peroxidation. Importantly, simultaneous SPARC knockdown attenuated these effects (Figure 6L,M and Figure S4B–D). Conversely, CUX1 overexpression in HG‐treated podocytes reversed mitochondrial dysfunction and ferroptosis (Figure 6N,O and Figure S4E–G). To link CUX1 with Klotho, IF, Western blot, and IHC analyses revealed a positive correlation between CUX1 and Klotho expression in podocytes across DKD mouse models, which was supported by in vitro experiments (Figure 6P–R and Figure S4H). These findings demonstrate that CUX1 acts as the key transcriptional repressor through which Klotho suppresses SPARC expression, thereby protecting podocytes from ferroptosis and mitochondrial damage in DKD.

### 2.6 PKCα Drives CUX1 Degradation in the Context of Reduced Klotho

**FIGURE 7:**
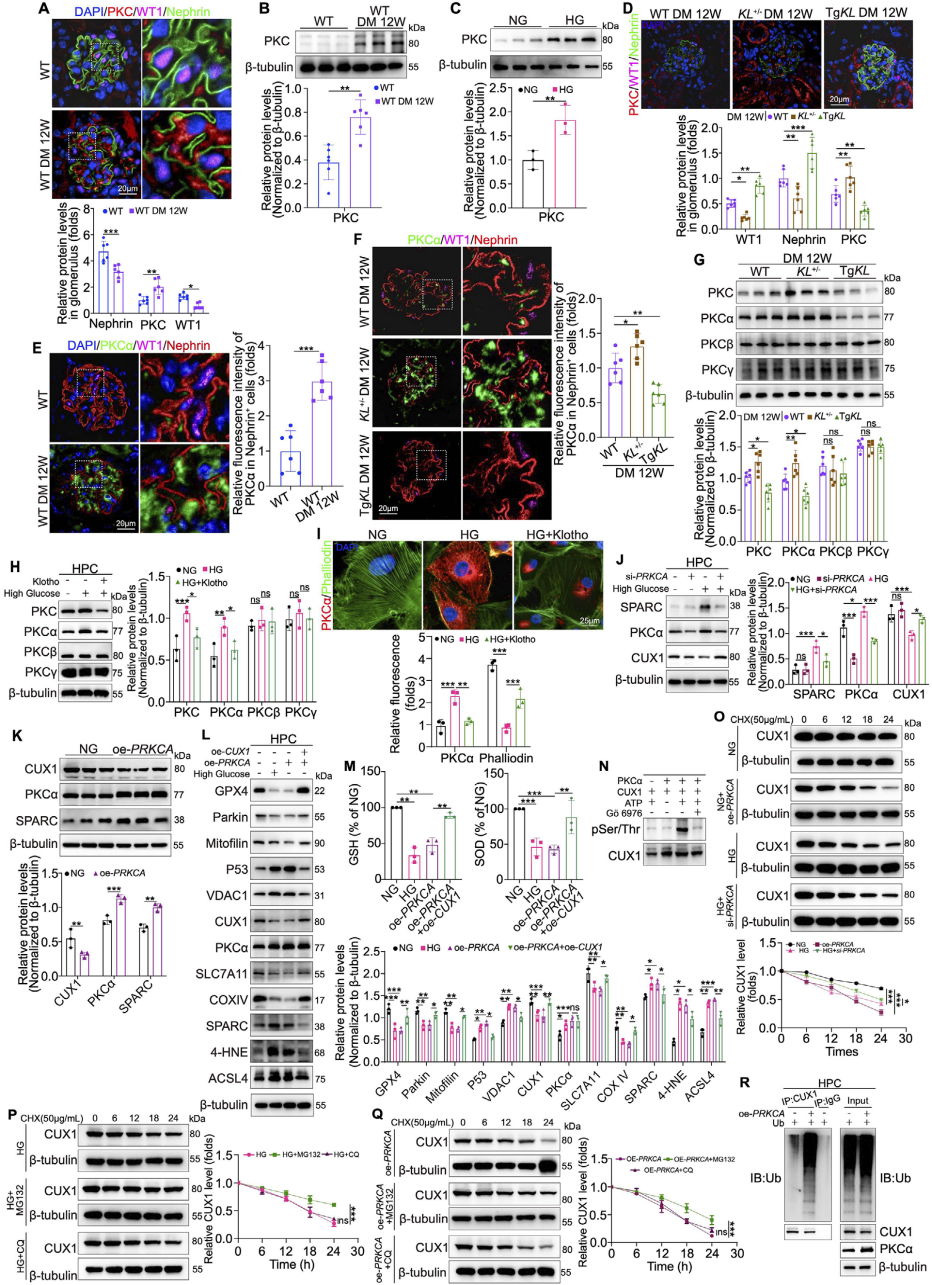
PKCα promotes CUX1 degradation under Klotho‐deficient conditions. (A) Co‐IF staining and quantitative analysis of PKC, Nephrin, and WT1 in glomeruli of control and diabetic mice (*n* = 6). Scale bar, 20 µm. (B, C) Western blot and quantification analysis of PKC expression in isolated glomeruli of diabetic mice (B, *n* = 6) and in podocytes under HG (C, *n* = 3). (D) Co‐IF staining and quantitative analysis of PKC, Nephrin, and WT1 in kidney sections from WT, *KL* ^+/−^, and Tg*KL* diabetic mice (*n* = 6). Scale bar, 20 µm. (E, F) IF staining of PKCα, Nephrin, and WT1 and quantitative analysis of PKCα in podocytes in indicated mouse groups (*n* = 6). Scale bar, 20 µm. (G, H) Protein levels and quantification of PKCα, PKCβ, and PKCγ in mouse isolated glomeruli and podocytes (*n* = 6). (I) Representative images and quantification of PKCα and Phalloidin in podocytes pretreated with recombinant Klotho before HG exposure (*n* = 3). Scale bar, 25 µm. (J, K) Western blot analysis and quantification of CUX1 and SPARC expression in podocytes following PKCα knockdown (J) or overexpression (K) (*n* = 3). (L) Western blot and quantification analysis of oxidative stress‐ and mitochondrial‐related proteins (GPX4, SLC7A11, Parkin, Mitofilin, COXIV, VDAC1, P53, 4‐HNE, ACSL4) and CUX1/SPARC/PKCα in podocytes with PKCα overexpression ± CUX1 co‐expression (*n* = 3). (M) PKCα overexpression reduced GSH and SOD levels, similar to HG treatment, an effect reversed by CUX1 overexpression (*n* = 3). (N) Recombinant CUX1 was incubated with PKCα, ATP, and Gö 6976 as indicated. Phosphorylation (pSer/Thr) and total CUX1 (loading control) were detected by immunoblotting. (O–Q) Podocytes were treated with PKCα modulator, HG, CHX, and either MG132 or CQ as indicated, then CUX1 protein stability was assessed (*n* = 3). (R) Podocytes were transfected with oe‐PKCα and Ub as indicated. Co‐IP with anti‐CUX1 antibody followed by anti‐Ub blotting showed that PKCα overexpression increased the ubiquitination of CUX1. Data are presented as mean ± SD; ns, not significant; ^*^ *p* < 0.05, ^**^ *p* < 0.01, ^***^ *p* < 0.001, One‐way ANOVA with Dunnett's test or Tukey's test (D, F–J, L–M), Two‐way ANOVA with Dunnett's test or Tukey's test (O–Q), unpaired Student's *t*‐test (A–C, E and K). IF, Immunofluorescence; HG, high glucose; WT, Wild‐Type; CQ, chloroquine; Ub, Ubiquitin; CHX, Cycloheximide.

Since Klotho is neither a kinase nor a transcription factor, its regulation of CUX1 likely involves an intermediary. Notably, CUX1's DNA binding and transcriptional activity are known to be regulated by post‐translational modifications, including phosphorylation by protein kinase C (PKC). Our prior work also suggested that Klotho modulates PKC expression. We therefore investigated whether PKC mediates Klotho's effect on CUX1 [33, 34]. IF staining of podocytes in diabetic mice at week 12 post‐induction revealed significantly elevated total PKC levels (Figure 7A), confirmed by Western blot in glomerular tissue and cultured podocytes (Figure 7B,C). Among PKC isoforms (α, β, γ), only PKCα showed a strong negative correlation with Klotho and accounted for the majority of PKC upregulation within DKD glomeruli (Figure 7D–G and Figure S5A).

In vitro, HG stimulation of podocytes induced PKCα upregulation, which was effectively suppressed by Klotho overexpression, as demonstrated by both Western blot and IF staining (Figure 7H,I). To determine whether PKCα regulates CUX1 and thereby affects SPARC transcription, we knocked down PKCα expression using siRNA in HG‐treated podocytes. This intervention significantly restored CUX1 protein levels while reducing SPARC expression (Figure 7J). Moreover, PKCα inhibition reversed key features of mitochondrial dysfunction and ferroptosis: it enhanced intracellular GSH content and SOD activity; increased the expression of SLC7A11, COXIV, GPX4, Mitofilin, and Parkin; and decreased P53, 4‐HNE, and VDAC1. Functionally, PKCα blockade preserved mitochondrial membrane potential (JC‐1 staining), suppressed mitochondrial ROS production (MitoSOX), and ameliorated mitochondrial fragmentation (MitoTracker) (Figure S5B–D).

Conversely, forced overexpression of PKCα recapitulated the effects of HG, inducing profound mitochondrial damage and ferroptotic signaling. Specifically, PKCα overexpression suppressed SLC7A11, GPX4, other mitochondrial membrane proteins, COXIV, Mitofilin, and Parkin, while elevating P53, VDAC1, SPARC, 4‐HNE, and ACSL4, accompanied by diminished GSH levels and reduced SOD activity (Figure 7K–M). Functionally, this led to collapse of the mitochondrial membrane potential, increased mitochondrial ROS generation, exacerbated mitochondrial fragmentation, and elevated lipid peroxidation (Figure S5E). Strikingly, co‐overexpression of CUX1 under these conditions substantially rescued the pathological phenotype, suggesting that exogenous CUX1 compensates for its PKCα‐mediated depletion (Figure 7L,M, and Figure S5E). Mechanistic studies demonstrated that PKCα directly phosphorylates CUX1, as shown by in vitro kinase assays, and this phosphorylation was inhibited by a PKCα inhibitor (Figure 7N). Cycloheximide (CHX) chase assays revealed that PKCα overexpression markedly shortened CUX1 half‐life, indicating active promotion of CUX1 degradation (Figure 7O). Using the proteasome inhibitor MG132 and lysosome inhibitor chloroquine, we found that MG132, but not chloroquine, prevented CUX1 degradation, confirming that PKCα promotes proteasome‐mediated degradation of CUX1 (Figure 7P,Q). Consistently, PKCα overexpression increased CUX1 ubiquitination (Figure 7R). Taken together, these results define a molecular cascade in DKD in which Klotho deficiency induces PKCα upregulation, leading to phosphorylation and subsequent degradation of CUX1. Loss of CUX1 derepresses SPARC, triggering mitochondrial dysfunction and ferroptosis in podocytes.

## 3 Discussion

DKD is a major cause of ESRD, which frequently necessitates renal replacement therapies such as dialysis or kidney transplantation [1, 35]. Understanding the mechanisms that drive DKD onset and progression is therefore critically important. Podocytes, highly specialized epithelial cells essential for maintaining the integrity of the filtration barrier, represent key cellular targets in DKD. Injury, dysfunction, and eventual depletion of podocytes are central events in DKD pathogenesis and strongly correlate with disease severity [36, 37]. Ferroptosis, a form of regulated cell death driven by iron‐dependent lipid peroxidation and metabolic disturbances, has gained increasing attention in kidney diseases. Accumulating evidence implicates podocyte ferroptosis as a pivotal mechanism in glomerular damage during DKD [38, 39, 40]. In this study, we uncover a novel transcriptional mechanism by which Klotho confers podocyte protection under DKD conditions. Although Klotho is predominantly expressed in renal tubular epithelial cells, its detectable expression in podocytes suggests that Klotho may also exert local protective effects within the glomerulus, either through direct intracellular actions or through autocrine and paracrine mechanisms [29, 41]. Regarding the regulatory mechanisms of Klotho in podocytes during DKD, we demonstrate that Klotho maintains the stability of the transcription factor CUX1, thereby enabling repression of SPARC transcription. The CUX1/SPARC axis functions as a decisive molecular switch that activates the TGFβ‐RII/Smad signaling pathway, driving mitochondrial dysfunction and ferroptosis in podocytes. Furthermore, we identified that PKCα acts as a critical upstream amplifier of this cascade. Klotho deficiency increases PKCα expression, which phosphorylates CUX1 and accelerates its subsequent degradation, further elevating SPARC levels and exacerbating podocyte mitochondrial dysfunction and ferroptosis. Together, our findings establish SPARC as a central effector of podocyte mitochondrial damage and ferroptosis in DKD and define a previously unrecognized Klotho/PKCα/CUX1/SPARC/TGFβ‐RII signaling network. Importantly, this study highlights CUX1‐mediated repression of SPARC as a critical protective checkpoint against ferroptosis, providing mechanistic insight into DKD progression and identifying SPARC as a potential therapeutic target to preserve podocyte integrity and slow disease progression.

Secreted protein acidic and rich in cysteine is a multifunctional matricellular protein that regulates adhesion, motility, and proliferation via cell‐cell and cell‐matrix interactions and has been implicated in TGFβ1‐driven tumor progression and hypertension [27, 42, 43]. Notably, SPARC can selectively co‐opt the TGFβ signaling system to suppress epithelial cell proliferation by engaging TGFβ receptors and activating the Smad2/3 pathway [44]. A recent study reported that SPARC regulates ferroptosis in hepatocellular carcinoma cells under sorafenib treatment, while in myocardial infarction, SPARC has been shown to induce secondary mitochondrial dysfunction [28]. Our work now extends these observations to the kidney, revealing that SPARC is markedly overexpressed in DKD and drives podocyte mitochondrial dysfunction and ferroptosis through TGFβ‐RII/Smad activation. Notably, circulating SPARC levels were elevated in DKD mice, correlating with glomerular SPARC expression across genotypes (WT, *KL* ^+/−^, Tg*KL*), suggesting potential as a noninvasive biomarker for podocyte injury, warranting further clinical investigation. Beyond DKD, our previous work demonstrated that SPARC promotes renal tubular fibrosis in nondiabetic injury models such as UUO and IRI [45]. Here, we further show that SPARC deletion also attenuates podocyte ferroptosis in models such as UUO and IRI, suggesting that its function extends beyond fibrotic remodeling to broader regulation of mitochondrial dysfunction and ferroptosis in the kidney. The context‐dependent mechanisms of SPARC across different renal compartments, however, remain to be fully defined.

DKD progression is increasingly understood as driven by self‐amplifying networks in which mitochondrial dysfunction and ferroptosis reinforce each other [18, 46]. Our study found that SPARC induction following Klotho deficiency initiates a cascade via PKCα‐mediated CUX1 degradation, promoting mitochondrial damage and ferroptosis. PKCα, the predominant PKC isoform implicated in DKD, is activated by metabolic stressors, whereas PKCβ mainly contributes to immune‐mediated glomerulonephritis [47, 48, 49, 50]. Our results show that Klotho selectively and significantly suppresses PKCα, though the precise mechanism remains to be defined, possibly involving modulation of intracellular calcium [34, 51]. Finally, this study has several considerations. First, because Klotho and SPARC are not podocyte‐specific, our global models do not fully resolve cell‐type‐specific contributions. However, consistent phenotypes observed in isolated podocytes, the protective effects of recombinant Klotho, and the in vivo spatial association of SPARC with podocyte injury collectively support a dominant podocyte‐intrinsic role of this pathway. Second, while SPARC deletion showed clear protective effects, contributions from other renal compartments cannot be excluded. Future studies using conditional, cell‐specific knockout models will help further refine these effects. Third, although PKCα‐mediated phosphorylation and enhanced CUX1 degradation were demonstrated, the absence of direct E3 ligase activity suggests that additional ubiquitination machinery is involved, which remains to be identified. Finally, the human cohort is relatively small and cross‐sectional, limiting causal inference and clinical generalizability.

In summary, our study reveals a Klotho/PKCα/CUX1/SPARC/TGFβ‐RII axis linking mitochondrial dysfunction and ferroptosis in podocytes. Targeting this pathway, especially SPARC, may offer a promising approach to protect podocytes and slow DKD progression.

## 4 Materials and Methods

### 4.1 Human Samples

This study was approved by the Ethics Committee of the Children's Hospital of Chongqing Medical University (Ethics Approval No. 2025442) and conducted in accordance with the Declaration of Helsinki. This study enrolled patients with DM at the Affiliated Hospital of North Sichuan Medical College, with approval from its Ethics Committee (File Number: 2025ER443‐1). Patients met the diagnostic criteria of the 2024 American Diabetes Association guidelines. Relevant clinical characteristics of the participants are provided in Table S1. Six healthy controls were also included; control kidney tissues were obtained from histologically normal renal cortex distal to the tumor in patients undergoing nephrectomy for non‐renal malignancies. Core needle biopsy specimens were collected following written informed consent from patients or guardians, based on clinical indications determined by the attending nephrologist. Each sample was independently evaluated by two board‐certified nephropathologists using stereomicroscopy and electron microscopy to ensure diagnostic accuracy and consistency.

### 4.2 Animal Models

Eight‐week‐old male C57BL/6J mice were purchased from Beijing HFK Biotechnology Co., Ltd. Klotho‐overexpressing transgenic (Tg*KL*) and heterozygous Klotho‐deficient (*KL* ^+/−^) mice, maintained on a C57BL/6 background, were generated in our laboratory as previously described [23]. *SPARC* knockout (*SPARC* ^−/−^) mice were obtained from Shanghai Model Organisms Center Inc. Genotyping was performed by PCR using tail genomic DNA (primers in Table S2). DKD was induced in WT, *KL* ^+/−^, and Tg*KL* mice via intraperitoneal injections of streptozotocin (STZ, 55 mg/kg in 0.1 M sodium citrate, pH 4.5) for seven consecutive days, and then they were provided with a high‐fat diet (HFD) for one month. Control mice received vehicle alone. Mice with fasting blood glucose > 16.7 mM one week after the final injection were considered diabetic. Additional kidney injury models included: (1) UUO: WT and *SPARC* ^−/−^ mice were anesthetized with pentobarbital (50 mg/kg, *i.p*.), and the left ureter was ligated at two sites. (2) IRI: The left renal pedicle was clamped for 30 min to induce ischemia, followed by reperfusion. All mice were maintained under SPF conditions at the Laboratory Animal Research Center, Children's Hospital of Chongqing Medical University. Protocols adhered to NIH guidelines and were approved by the Institutional Animal Welfare and Ethics Committee (Approval No. CHCMU‐IACUC20250804008).

### 4.3 Isolation of Glomeruli

Isolation of glomeruli was performed using magnetic beads‐based separation with the following optimized steps. First, the kidneys were perfused with Dynabeads M‐450 Tosylactivated (Invitrogen) to allow bead entrapment within glomerular capillaries. The renal cortex was then dissected and minced into small fragments. Tissue digestion was carried out in a mixture containing 1 mg/mL collagenase A and 100 U/mL DNase I at 37°C for 30 min under gentle agitation. After digestion, the tissue suspension was sequentially passed through 100 and 70 µm cell strainers. The filtrate was collected and washed using magnetic separation with a magnetic stand. The bead‐bound glomeruli were retained.

### 4.4 Cell Culture

HEK293T cells were cultured in DMEM supplemented with 10% fetal bovine serum (FBS) and 1% penicillin/streptomycin at 37°C in 5% CO_2_. Conditionally immortalized human podocytes were maintained at 33°C in RPMI‐1640 containing 10% FBS, 1% Insulin‐Transferrin‐Selenium (ITS), and 1% Penicillin/ Streptomycin. Differentiation was induced by transferring cells at 70%–80% confluence to 37°C in ITS‐free RPMI‐1640 for 14 days, confirmed by podocyte‐specific marker expression. Differentiated podocytes were serum‐starved for 6 h and treated with 30 mM D‐glucose for 48 h with SPARC (1 µg/mL, HY‐P7291, MCE Biotech), Fer‐1 (5 µM, HY‐100579, MCE Biotech), TGFβ RII‐IN‐3 (5 µM, HY‐161398, MCE Biotech) or preincubated with Klotho (400 pM, ab84072, Abcam). The 30 mM glucose concentration was selected based on our previous study using the identical podocyte system, in which hyperosmolarity effects had been excluded [34].

### 4.5 Determination of Renal Function

After weighing the animals, urine samples were collected after centrifugation (15 min, 3500 rpm, 4°C) and stored at ‐20°C until they were tested. Urine protein and creatinine (Cr) were determined using a urine protein test kit and a creatinine assay kit (C035‐2‐1, C011‐2‐1, Nanjing Jiancheng Bioengineering Institute). The blood was centrifuged at 3000 rpm for 10 min to collect serum. The concentrations of serum creatinine and BUN were measured in serum using the creatinine assay kit and urea assay kit (C011‐2‐1, C013‐2‐1, Nanjing Jiancheng Bioengineering Institute) according to the manufacturer's instructions, respectively.

### 4.6 ELISA

Mouse blood was centrifuged (1000×g, 20 min), and supernatants were stored at −80°C. Serum SPARC was measured using a mouse osteonectin ELISA kit (CSB‐E08762m, CUSABIO), following the manufacturer's instructions.

### 4.7 TEM

Mouse kidneys were cut into 1 mm sections and fixed in 2.5% glutaraldehyde. Ultrastructural analysis, including mitochondrial morphology, basement membrane integrity, and podocyte foot process architecture, was performed at the Electron Microscopy Core Laboratory of Chongqing Medical University.

### 4.8 Detection of C11‐BODIPY581/591 and Fe^2+^

For lipid peroxidation, podocytes were washed with PBS and stained with C11‐BODIPY^5^ ^8^ ^1^/^5^ ^9^ ^1^ (HY‐D1301, MCE; 1 µM) in serum‐free medium at 37°C for 30 min. After staining, cells were washed twice with PBS. To detect intracellular Fe^2^^+^, cells were incubated with FerroOrange (F374, Dojindo; 1 µM) at 37°C for 30 min. Fluorescence images were acquired using an inverted fluorescence microscope.

### 4.9 Mitochondrial Function

Mitochondrial morphology was assessed by staining cells with MitoTracker Red (C1035, Beyotime) for 30 min in the dark. Mitochondrial membrane potential was evaluated using JC‐1 dye (C2006, Beyotime), and mitochondrial reactive oxygen species (mtROS) were detected with MitoSOX Red (GR‐30921, Yeasen), while the ATP content was measured using an ATP Assay Kit (S0026, Beyotime), all according to the manufacturers’ protocols. Fluorescent images were acquired using a Nikon A1R Meta confocal microscope.

### 4.10 Tissue Analyses

Kidney tissues were fixed in 10% formalin, dehydrated in 70% ethanol, paraffin‐embedded, and sectioned at 3 µm for H&E and PAS (G1120, Solarbio) staining. For immunohistochemistry (IHC), sections were deparaffinized, rehydrated, and subjected to antigen retrieval using high‐pressure heating in citrate buffer (pH 6.0; ZLI‐9064, ZSGB‐BIO) or Tris–EDTA buffer (pH 9.0; ZLI‐9072, ZSGB‐BIO) for 10 min. Endogenous peroxidase was blocked with 3% H_2_O_2_ for 10 min. Sections were incubated with primary antibodies for 1 h at room temperature, followed by HRP‐conjugated secondary antibodies for 20 min. Signals were developed with DAB (DA1010, Solarbio), counterstained with hematoxylin, and imaged on an Olympus light microscope. For IF, sections were blocked with 10% goat serum for 1.5 h, incubated with primary antibodies overnight at 4°C, then with fluorophore‐conjugated secondary antibodies. Nuclei were labeled with DAPI (1:1000; C1002, Beyotime) for 10 min, and images were acquired using a Nikon A1R Meta confocal microscope. Primary antibodies are listed in Table S3.

### 4.11 Measurement of Antioxidant Enzyme Activity

GSH levels in human podocytes lysates were measured using a GSH‐Px Assay Kit (A005‐1‐2, Nanjing Jiancheng Bioengineering Institute). Absorbance at 412 nm was used to calculate GSH concentration, normalized to total protein content. Total SOD activity was assessed with a WST‐8–based SOD Assay Kit (S0101M, Beyotime) per the manufacturer's instructions. Absorbance at 450 nm was recorded, and SOD activity was expressed relative to protein concentration.

### 4.12 siRNA Knockdown

Gene silencing of *SPARC*, *CUX1* and *PRKCA* was achieved using small interfering RNA (siRNA) purchased from Gene Create. For each gene, three different siRNA sequences were initially screened. The most effective sequence, which was validated by qPCR to reduce target mRNA expression by more than 80%, was selected for all subsequent experiments. The final selected sequences are given in the Table S4.

### 4.13 Plasmid Construction

All plasmid vectors (pGL3‐Basic, pcDNA3.1‐HA, pcDNA3.1‐Flag) were obtained from Addgene. Truncated and wild‐type versions of the *SPARC* promoter were cloned into the pGL3‐Basic vector to generate luciferase reporter constructs. For transcription factor overexpression, the coding sequences of *CUX1* and *PRKCA* were cloned into the pcDNA3.1 vector. Plasmids HA‐Ub were provided by Obio Technology. All constructed plasmids were verified by DNA sequencing.

### 4.14 Transient Transfection

siRNA for target genes or negative control siRNA, empty vector control plasmid, or plasmid containing target genes were delivered into cells by the Lipofectamine 3000 (L3000015, ThermoFisher) following the manufacturer's protocol. After 6 h of transfection, the medium was replaced with fresh medium containing 10% FBS and cells were cultured for an additional 48 h.

### 4.15 Dual‐Luciferase Reporter Assay

HEK293T cells were co‐transfected with the CUX1 overexpression plasmid together with either the wild‐type SPARC promoter‐pGL3‐Basic or the mutant SPARC promoter construct. At 48 h post‐transfection, luciferase activity was measured using a commercial kit (11402ES60, Jiangsu Keygen Biotech) according to the manufacturer's instructions.

### 4.16 Co‐Immunoprecipitation

HPC cells were lysed in pre‐chilled mild lysis buffer supplemented with protease inhibitor cocktail (EHY‐K0010, MCE Biotech). For each immunoprecipitation, 1 mg of total protein lysate was incubated with the appropriate primary antibody (anti‐TGFBR2, 66636‐1‐Ig, Proteintech, anti‐SPARC, 15274‐1‐AP, Proteintech or anti‐CUX1, 11733‐1‐AP, Proteintech) overnight at 4°C to form antigen–antibody complexes. These complexes were captured by adding Protein A/G magnetic beads (B23202, Selleck) and incubating for 12 h at 4°C with gentle rotation. After extensive washing, bound proteins were eluted in SDS‐PAGE loading buffer under denaturing conditions and analyzed by western blotting.

### 4.17 PLA

Following differentiation of HPCs on glass coverslips, the cells were subjected to high‐glucose treatment. Fixation was carried out using 4% paraformaldehyde, and permeabilization was achieved by applying the Duolink blocking reagent. Subsequently, primary antibodies recognizing SPARC and TGFβ‐RII were prepared in the blocking buffer and added onto the coverslips. Detection was performed by incubating the samples with one anti‐rabbit PLUS and one anti‐mouse MINUS PLA probe (Sigma–Aldrich, DUO92002 and DUO92004, respectively). The ligation and rolling‐circle amplification reactions were conducted as detailed in the product manual (Sigma–Aldrich, DUO92013). Coverslips were then sealed with Duolink in Situ Mounting Medium supplemented with DAPI (Sigma–Aldrich, DUO82040). All images were captured on a Nikon A1 confocal microscope.

### 4.18 CHX Chase Assay

To monitor the half‐life of CUX1 protein, CHX chase assays were performed in podocytes. After stimulation with high glucose or transfection with si‐*PRKCA* or *PRKCA*‐overexpressing plasmid, cells were treated with 50 µg/mL CHX (S7418, Selleck) and collected at indicated time points (0, 6, 12, 18, 24 h). To identify the relevant proteolytic pathway, cells were co‐treated with either the proteasome inhibitor MG132 (10 µM, S2619, Selleck) or the lysosome inhibitor chloroquine (CQ, 50 µM, S6999, Selleck). CUX1 protein levels at each time point were quantified by western blotting.

### 4.19 In Vitro Kinase Assay

To determine whether PKCα directly phosphorylates the transcription factor CUX1, an in vitro kinase assay was performed. The reaction mixture contained 50 mM Tris‐HCl (pH 7.5), 1 mM DTT, 10 mM MgCl_2_, 0.1 mg/mL phosphatidylserine (PS), 10 µg/mL diacylglycerol (DAG), 0.1 mM CaCl_2_, 10 µM ATP, 100 ng of recombinant human active PKCα protein (ab55672, Abcam), and 2 µg of recombinant human CUX1 protein (PROTQ13948, Boster Biotech). To confirm the specificity of PKCα, Gö 6976 (S7119, Selleck) was added to the reaction mixture. The reaction mixture was incubated at 30°C for 30 min, and the reaction was terminated by adding 5× reducing SDS‐PAGE loading buffer followed by boiling for 5 min. Proteins were then separated by SDS‐PAGE, and Western blotting was performed using an anti‐Phospho—(Ser/Thr) (ab300625, Abcam). The total CUX1 antibody (11733‐1‐AP, Proteintech) was used to assess loading.

### 4.20 LC‐MS/MS Analysis

Cell pellets (≥ 1 × 10^6^ cells per sample) were processed for liquid extraction and analyzed by liquid chromatography–tandem mass spectrometry (LC‐MS/MS) at PTM BioLab, Inc. Tryptic peptides were reconstituted and separated on a custom‐made reversed‐phase analytical column. Eluted peptides were ionized via a capillary ion source and analyzed using a timsTOF Pro mass spectrometer (Bruker).

### 4.21 Western Blot Analysis

Proteins were extracted from renal tissues, isolated glomeruli, and cultured cells using RIPA lysis buffer (WB3100, NCM Biotech) supplemented with protease inhibitor cocktail (EHY‐K0010, MCE Biotech) and phosphatase inhibitor cocktail (C0001, TargetMol). Equal amounts of protein, as determined by concentration assay, were separated by SDS‐PAGE and transferred onto PVDF membranes. Membranes were blocked with 5% (w/v) skim milk in TBST for 1.5 h at room temperature, then incubated overnight at 4°C with specific primary antibodies. After three TBST washes, membranes were probed with HRP‐conjugated secondary antibodies for 1 h at room temperature. Immunoreactive bands were detected using enhanced chemiluminescence substrate (Pierce ECL, ThermoFisher Scientific) and quantified by densitometry using ImageJ software. Primary antibodies are listed in Table S3.

### 4.22 qPCR Analysis

Total RNA was extracted from cells using the SteadyPure Quick RNA Extraction Kit (AG21023, Accurate Biology). Complementary DNA (cDNA) was synthesized from 1 µg of total RNA using the Evo M‐MLV RT Kit (CAG11708, Accurate Biology) following the manufacturer's protocol. Quantitative real‐time PCR was performed with SYBR Green Master Mix (AG11761, Accurate Biology) on a Thermo Fisher Scientific real‐time PCR system. Gene expression levels were normalized to GAPDH and analyzed using the 2−ΔΔCt method. Primer sequences are listed in Table S5.

### 4.23 Statistical Analysis

All data are presented as mean ± SD from at least three independent experiments. Statistical analyses were performed using GraphPad Prism 10.0 software. The correlation analysis was examined using Pearson's correlation coefficients. Differences between two groups were evaluated by Student's *t*‐test, while multiple group comparisons were conducted by one‐way ANOVA. *p* < 0.05 was considered statistically significant, and significance levels are denoted as ^*^ *p* < 0.05, ^**^ *p* < 0.01, and ^***^ *p* < 0.001. The sample size (n) for each experiment was provided in the figure legends.

## Author Contributions

H.P.Y., W.J. and Q.L. conceived and designed the study. Q.Y. and H.M.J. performed the experiments and drafted the manuscript. C.Y.X., X.D.Z., Q.Q.T., H.X., L.Q., and C.G. collected and analyzed the clinical data. Y.X.C. provided critical revisions for important intellectual content. All authors had full access to the study data, reviewed manuscript drafts, and approved the final version. Q.Y. and H.M.J., contributed equally to this work.

## Funding

This study was supported by the National Natural Science Foundation of China (No. 82200906 and 82170720), the Chongqing Natural Science Foundation (No. CSTB2025NSCQ‐GPX1191 and CST B2024NSCQ‐MSX0422), the Joint Medical Research Programs of Chongqing Science and Technology Bureau and Health Commission Foundation (No. 2023GGXM001 and 2025QNXM03 9), and the Western China Birth Cohort (No. NCRCCHD‐2023‐MP‐01).

## Ethics Statement

This study was conducted in accordance with the Declaration of Helsinki. The collection and use of human renal biopsy specimens were approved by the Ethics Committee of the Children's Hospital of Chongqing Medical University (Approval No. 2025442) and the Ethics Committee of the Affiliated Hospital of North Sichuan Medical College (Approval No. 2025ER443‐1). All patients or their guardians provided written informed consent prior to biopsy collection. All animal experiments were performed in accordance with the National Institutes of Health (NIH) guidelines for the care and use of laboratory animals. Animal protocols were approved by the Institutional Animal Welfare and Ethics Committee of the Children's Hospital of Chongqing Medical University (Approval No. CHCMU‐IACUC20250804008). All mice were maintained under specific pathogen‐free (SPF) conditions, and all procedures were designed to minimize animal suffering.

## Conflicts of Interest

The authors declare that they have no conflicts of interests.

## Supporting information




**Supporting File**: advs77202‐sup‐0001‐SuppMat.docx.

## Data Availability

All data and materials used in this study are available from the corresponding author upon reasonable request.
