# Supplementary material for: SPARC Drives Podocyte Mitochondrial Damage and Ferroptosis in Diabetic Kidney Disease Following Klotho Deficiency
Source: Adv Sci (Weinh). 2026 Aug 29:e77202. Online ahead of print. doi: 10.1002/advs.77202 (PMC13525411; doi:10.1002/advs.77202)
Supplement: Supplementary file 1 [file ADVS-9999-e77202-s001.docx]

**Supplemental Figure 1. Klotho deficiency exacerbates podocyte injury.** (A) Fasting blood glucose, BUN, serum creatinine, and UACR were measured in different groups (n=6-11). (B) Representative HE and PAS staining of kidney sections from control and diabetic mice (n=6). Scale bar, 50 µm. (C) TEM images showing glomerular basement membrane thickening and foot process effacement (n=8-10). Scale bar, 1 µm. (D) Immunohistochemical detection and quantitative analysis of Klotho in glomeruli of control and diabetic mice (n=6). Scale bar, 50 µm. (E) Immunofluorescence co-staining of 4-HNE and Nephrin and quantitative analysis of 4-HNE in podocytes in kidney sections from WT and WT DM 12W mice (n=6). Scale bar, 20 µm. (F) Detection of cellular ferrous iron levels using FerroOrange fluorescent and quantitative analysis probe in three different treatment groups (n=3). Scale bar, 25 µm. (G) Western blot and quantitative analysis of renal Klotho expression in mice (n=6). (H) BUN, serum creatinine, and UACR were measured in different groups (n=8-10). (I) HE and PAS staining of kidney sections from WT, *KL*^+/-^, and Tg*KL* diabetic mice (n=6). Scale bar, 50 µm. (J) Co-IF staining of 4-HNE and Nephrin and quantitative analysis of 4-HNE in podocytes in kidney sections from WT, *KL*^+/-^, and Tg*KL* diabetic mice (n=6). Scale bar, 20 µm. (K) Representative images of GPX4, SLC7A11 and Phalloidin staining and quantitative analysis in podocytes pretreated with recombinant Klotho before HG exposure (n=3). Scale bar, 25 µm. (L) SOD activity, GSH and ATP levels in podocytes under HG ± recombinant Klotho. (M) TEM images of podocyte mitochondria under indicated conditions (n=3). Scale bar, 1 µm. (N) FerroOrange and C11-BODIPY^581/591^ staining and quantitative analysis in HG, and HG+Klotho groups (n=3). Scale bar, 25 µm. Data are presented as mean ± SD; ^*^*P* < 0.05, ^**^*P* < 0.01, ^***^*P* < 0.001, One-way ANOVA with Dunnett's test or Tukey's test (F-H, J-L and N), unpaired Student's t-test (A, C-E). IF, Immunofluorescence; TEM, Transmission electron microscopy; HG, high glucose; WT, Wild-Type.

**Supplemental Figure 2. SPARC promotes ferroptosis in IRI and UUO.** (A) Quantitative analysis for FerroOrange in podocytes under indicated conditions (n=3). (B) IF staining and quantitative analysis for JC-1, MitoSOX, MitoTracker, GPX4, SLC7A11, C11-BODIPY^581/591^ in podocytes under indicated conditions (n=3). Scale bar, 25 µm. (C) Quantitative analysis for FerroOrange and C11-BODIPY^581/591^ in podocytes under indicated conditions (n=3). (D) BUN and serum creatinine were measured in mice subjected to IRI or UUO (n=6). (E, F) Representative HE, PAS, and SPARC IHC staining of mouse kidneys after IRI or UUO (n=6). Scale bar, 50 µm. (G) Glomerular co-IF staining for Nephrin, WT1, and SPARC and quantitative analysis of SPARC in podocytes in kidneys from mice subjected to IRI or UUO. (H) BUN and serum creatinine in WT and *Sparc*^-/-^ mice under IRI or UUO protocols (n=6). (I, J) HE and PAS analyses showing that SPARC knockdown attenuates renal injury in IRI and UUO models (n=6). Scale bar, 50 µm. Data are presented as mean ± SD; ^*^*P* < 0.05, ^**^*P* < 0.01, ^***^*P* < 0.001, One-way ANOVA with Dunnett's test or Tukey's test (A-C), unpaired Student's t-test (D, F-H). IF, Immunofluorescence; IHC, Immunohistochemistry; HG, high glucose; WT, Wild-Type.

**Supplemental Figure 3. SPARC knockout alleviates tubular ferroptosis in IRI and UUO.** (A, B) IF localization and expression of GPX4 and Nephrin and quantitative analysis of GPX4 in podocytes in kidney tissues from SPARC knockout mice following IRI and UUO mice (n=6). Scale bar, 20 µm. (C, D) IHC staining and quantitative analysis showing glomerular Beclin1 and MFN1 expression across mouse groups (n=6). Scale bar, 10 µm. (E, F) Western blot and quantification of SPARC, GPX4, Parkin, Mitofilin, 4-HNE, and COXIV in WT and SPARC knockout mice following IRI (E) or UUO (F) (n=6). (G, H) Co-IF staining and quantitative analysis of the tubular marker AQP1 with ferroptosis regulators SLC7A11 and GPX4 in kidney sections from WT and *SPARC*^-/-^ mice after IRI or UUO (n=6). Scale bar, 50 µm. Data are presented as mean ± SD; ^*^*P* < 0.05, ^**^*P* < 0.01, ^***^*P* < 0.001, One-way ANOVA with Tukey's test (A-H). IF, Immunofluorescence; IHC, Immunohistochemistry; HG, high glucose; WT, Wild-Type.

**Supplemental Figure 4. CUX1 mitigates mitochondrial damage in podocytes during DKD.** (A) IHC staining showing glomerular CUX1 expression and quantitative analysis of CUX1 in podocytes in control and diabetic mice (n=6). Scale bar, 50 µm. (B) IF staining and quantitative analysis showing increased SPARC-Phalloidin colocalization in podocytes upon CUX1 knockdown (n=3). Scale bar, 25 µm. (C) Western blot and quantitative analysis showing that CUX1 silencing reduced GPX4, Parkin, Mitofilin, and COXIV levels and increased p53, VDAC1, 4-HNE, ACSL4, and SPARC; these effects were reversed by SPARC knockdown (n=3). (D) IF staining reveals that CUX1 loss exacerbates mitochondrial damage, lipid peroxidation, and iron accumulation in podocytes, which is rescued by SPARC suppression (n=3). Scale bar, 25 µm. (E) Western blot and quantitative analysis showing protein levels of CUX1, ferroptosis- and mitochondrial-related markers (GPX4, SLC7A11, Parkin, Mitofilin, COXIV, P53, 4-HNE, VDAC1, SPARC) in podocytes under indicated treatments (n=3). (F, G) IF staining and quantitative analysis for SPARC (F), MitoTracker, JC-1, and MitoSOX (G) in podocytes under corresponding conditions (n=3). Scale bar, 25 µm. (H) Renal CUX1 expression is reduced in diabetic *KL*^+/-^ mice, linking Klotho deficiency to CUX1 downregulation (n=6). Scale bar, 50 µm. Data are presented as mean ± SD; ns, not significant; ^*^*P* < 0.05, ^**^*P* < 0.01, ^***^*P* < 0.001, One-way ANOVA with Dunnett's test or Tukey's test (C-H), unpaired Student's t-test (A, B). IF, Immunofluorescence; IHC, Immunohistochemistry; HG, high glucose; WT, Wild-Type.

**Supplemental Figure 5. PKCα exacerbates mitochondrial damage in podocytes in DKD.** (A) IHC staining and quantitative analysis of PKC isoforms (PKCα, PKCβ, PKCγ) in glomeruli across experimental groups (n=6). Scale bar, 50 µm. (B) PKCα knockdown partially restored HG-induced reductions in SOD and GSH in podocytes (n=3). (C) Western blot and quantitative analysis of mitochondrial and ferroptosis markers (GPX4, Parkin, Mitofilin, COXIV, P53, VDAC1, SLC7A11, 4-HNE) in podocytes under indicated treatments (n=3). (D) IF staining and quantitative (MitoTracker, JC-1, MitoSOX) shows that PKCα inhibition relieves HG-induced mitochondrial damage (n=3). Scale bar, 25 µm. (E) PKCα overexpression increases mitochondrial dysfunction and lipid peroxidation, an effect reversed by CUX1 co-expression (n=3). Scale bar, 25 µm. (F) PKCα overexpression reduced ATP levels, similar to HG treatment, an effect reversed by CUX1 overexpression (n=3). Data are presented as mean ± SD; ns, not significant; ^*^*P* < 0.05, ^**^*P* < 0.01, ^***^*P* < 0.001, One-way ANOVA with Tukey's test (A-F). IF, Immunofluorescence; IHC, Immunohistochemistry; HG, high glucose; WT, Wild-Type.

**Supplemental Table 1.** Clinical characteristics in DKD patients

Results are expressed as mean ± SD. DKD, diabetic kidney disease; GLD, glucose; Scr, serum creatinine; UPE, urine protein electrophoresis; eGFR, estimated glomerular filtration Rate; M, male; F, female.

| **Number** | **Age (year)** | **Sex** | **GLU (mmol/1)** | **Body Mass Index** | **Scr (μmol/1)** | **UPE/24h (g)** | **eGFR (ml/min/1.73m²)** |
| --- | --- | --- | --- | --- | --- | --- | --- |
| **1** | 44 | M | 5.6 | 25.3 | 122 | 3.1 | 61.5 |
| **2** | 56 | F | 10.9 | 31.3 | 118 | 5.88 | 44.5 |
| **3** | 52 | F | 6.0 | 27.5 | 161 | 4.5 | 31.3 |
| **4** | 45 | M | 11.1 | 30.3 | 154 | NA | 46.2 |
| **5** | 57 | F | 9.5 | 24.8 | 76 | 3.12 | 75.4 |
| **6** | 54 | M | 12.9 | 30.6 | 146 | 3.60 | 46.3 |
| **7** | 51 | F | 4.9 | 19.7 | 150 | 3.3 | 34.4 |
| **8** | 58 | M | 11.9 | 26.2 | 122 | 3.09 | 55.7 |
| **9** | 49 | F | 5.4 | 29.8 | 163 | 4.4 | 31.7 |
| **10** | 55 | M | 12.9 | 17.5 | 170 | 3.39 | 38.2 |
| **11** | 58 | M | 7.7 | 22.4 | 176 | 9.28 | 35.9 |
| **12** | 48 | F | 6.6 | 25.5 | 107 | 3.02 | 53.3 |
| **13** | 54 | M | 8.7 | 28.4 | 145 | 0.9 | 46.7 |
| **14** | 59 | F | 14.2 | 30.9 | 76 | 5.43 | 74.5 |
| **15** | 47 | F | 9.6 | 26.9 | 113 | 3.6 | 50.2 |

**Supplemental Table 2.** Primer sequences for genotyping

| **Name** | **Sequence Forward** | **Sequence Reverse** |
| --- | --- | --- |
| Tg*KL* PCR primer 1 | TTTGCCCTTTTTGAGTTT GGATCTT | GTGATGGGTGAAAGTGTCCCAGAT |
| Tg*KL* PCR primer 2 | ACCAAAAGCTGATAGAGGACAATG | GTGATGGGTGAAAGTGTCCCAGAT |
| Tg*KL* PCR primer 3 | GCAGAAGAGGACAGATACATTCAT | CCTACTG AAGAATCTATCCCACAG |
| *KL^+/-^* PCR primer 1 | TTGTGGAGATTGGAAGTGGACGAAAGAG | CTGGACCCCCTGAAGCTGGAGTTAC |
| *KL^+/-^* PCR primer 2 | TTGTGGAGATTGGAAGTGGACGAAAGAG | CGCCCCGACCGGAGCTGAGAGTA |
| *SPARC-*KO PCR primer 1 | TCTTGGAGTAAGACTGGAGCTGGG | GAAGAACCCACAGTAGACAATGGGC |
| *SPARC-*KO PCR primer 2 | CGGTCTCCCTTGGCATGAAAC | GCATGATGCTTCAGAAACCCAGA |

**Supplemental Table 3.** List of Primary antibodies

| **Primary antibody** | **Source** | **Provider** | **Catalog** | **Application** |
| --- | --- | --- | --- | --- |
| CUX1 | Rabbit | Proteintech | 11733-1-AP | IHC/IF (1:100), WB (1:1000),  IP (20ug/ml) |
| Nephrin | guinea pig | Progen | GP-N2 | IF (1:200) |
| WT1 | Mouse | Immunoway | YM6533 | IF (1:50) |
| WT1 | Rabbit | Abcam | Ab89901 | IF (1:50) |
| PKC | Rabbit | Abcam | Ab75991 | IHC/IF (1:400) |
| PKC | Rabbit | Abclonal | A24003 | WB (1:1000) |
| PKCα | Rabbit | Abclonal | A11107 | IHC/IF (1:100), WB (1:1000) |
| PKCβ | Rabbit | Abclonal | A21241 | IHC (1:100), WB (1:1000) |
| PKCγ | Rabbit | Abclonal | A9565 | IHC (1:1000), WB (1:1000) |
| MFN1 | Rabbit | Proteintech | 13798-1-AP | IHC/IF (1:100), WB (1:1000) |
| DRP1 | Rabbit | Proteintech | 12957-1-AP | WB (1:1000) |
| GPX4 | Rabbit | Abclonal | A25009 | IHC/ICC (1:50), WB (1:1000) |
| SLC7A11 | Rabbit | Proteintech | 26864-1-AP | IHC/ICC (1:100), WB (1:1000) |
| P53 | Rabbit | Proteintech | 10442-1-AP | WB (1:1000) |
| COXIV | Rabbit | Proteintech | 11242-1-AP | WB (1:1000) |
| Mitofilin | Rabbit | Proteintech | 10179-1-AP | WB (1:1000) |
| ACSL4 | Rabbit | Proteintech | 81196-1-RR | WB (1:1000) |
| Ubiquitin | Rabbit | Abclonal | A19686 | WB (1:2000) |
| VDAC1 | Rabbit | Proteintech | 55259-1-AP | WB (1:1000) |
| Smad2/3 | Rabbit | Abcam | AB202445 | WB (1:1000) |
| p-Smad2/3 | Rabbit | Abcam | AB254407 | WB (1:1000) |
| TGFBR2 | Rabbit | Invitrogen | PA5-35076 | IF (1:200), WB (1:1000) |
| TGFBR2 | Mouse | Proteintech | 66636-1-Ig | IP (20ug/ml) |
| Parkin | Rabbit | Immunoway | YM8468 | WB (1:1000) |
| PINK1 | Rabbit | Immunoway | YM8583 | IF (1:200), WB (1:3000) |
| Beclin1 | Rabbit | Proteintech | 11306-1-AP | IHC/ICC (1:100), WB (1:1000) |
| Klotho | Rabbit | Invitrogen | PA5-88303 | IHC (1:100), WB (1:1000) |
| SPARC | Rabbit | Abcam | ab225716 | WB (1:1000) for HPC |
| SPARC | Rabbit | Abcam | ab290636 | WB (1:1000) for mice |
| SPARC | Mouse | Proteintech | 66426-1-Ig | IHC (1:100) |
| SPARC | Rabbit | Proteintech | 15274-1-AP | IP (20ug/ml) |
| β-tubulin | Mouse | Proteintech | 66240-1-Ig | WB (1:3000) |

**Supplemental Table 4.** Primer sequences for siRNA

| **Name** | **Sense** | **Antisense** |
| --- | --- | --- |
| *SPARC* | AGACAGAGGUGGUGGAAGATT | UCUUCCACCACCUCUGUCUTT |
| *CUX1* | CGGCUUCUUCUACACACUGUUTT | AACAGUGUGUAGAAGAAGCCGTT |
| *PRKCA* | CCUUAUGUGAAGCUGAAACUA | UAGUUUCAGCUUCACAUAAGG |

**Supplemental Table 5.** Primer sequences for qPCR

| **Name** | **Sequence Forward** | **Sequence Reverse** |
| --- | --- | --- |
| *SPARC* | CCCATTGGCGAGTTTGAGAAG | CAAGGCCCGATGTAGTCCA |
| *GAPDH* | AGGTCGGTGTGAACGGATTTG | TGTAGACCATGTAGTTGAGGTCA |
